# Cek1 regulates ß(1,3)-glucan exposure through calcineurin effectors in *Candida albicans*

**DOI:** 10.1371/journal.pgen.1010405

**Published:** 2022-09-19

**Authors:** Andrew S. Wagner, Stephen W. Lumsdaine, Mikayla M. Mangrum, Ainsley E. King, Trevor J. Hancock, Timothy E. Sparer, Todd B. Reynolds

**Affiliations:** Department of Microbiology, University of Tennessee at Knoxville, Knoxville, Tennessee, United States of America; Duke University Medical Center, UNITED STATES

## Abstract

In order to successfully induce disease, the fungal pathogen *Candida albicans* regulates exposure of antigens like the cell wall polysaccharide ß(1,3)-glucan to the host immune system. *C*. *albicans* covers (masks) ß(1,3)-glucan with a layer of mannosylated glycoproteins, which aids in immune system evasion by acting as a barrier to recognition by host pattern recognition receptors. Consequently, enhanced ß(1,3)-glucan exposure (unmasking) makes fungal cells more visible to host immune cells and facilitates more robust fungal clearance. However, an understanding of how *C*. *albicans* regulates its exposure levels of ß(1,3)-glucan is needed to leverage this phenotype. Signal transduction pathways and their corresponding effector genes mediating these changes are only beginning to be defined. Here, we report that the phosphatase calcineurin mediates unmasking of ß(1,3)-glucan in response to inputs from the Cek1 MAPK pathway and in response to caspofungin exposure. In contrast, calcineurin reduces ß-glucan exposure in response to high levels of extracellular calcium. Thus, depending on the input, calcineurin acts as a switchboard to regulate ß(1,3)-glucan exposure levels. By leveraging these differential ß(1,3)-glucan exposure phenotypes, we identified two novel effector genes in the calcineurin regulon, *FGR41* and *C1_11990W_A*, that encode putative cell wall proteins and mediate masking/unmasking. Loss of either effector caused unmasking and attenuated virulence during systemic infection in mice. Furthermore, immunosuppression restored the colonization decrease seen in mice infected with the *fgr41Δ/Δ* mutant to wild-type levels, demonstrating a reliance on the host immune system for virulence attenuation. Thus, calcineurin and its downstream regulon are general regulators of unmasking.

## Author summary

*Candida albicans* is a common cause of nosocomial systemic infections, and has an attributed mortality rate as high as ~40–60%. Thus, disease management strategies are of high medical significance. One potential immunotherapeutic approach to control disease progression is to impact the ability of *C*. *albicans* to effectively evade host immune cells. This may be achieved by increasing the exposure of the highly immunogenic cell wall epitope ß(1,3)-glucan to the host immune system. Yet, elucidation of how ß-glucan exposure is regulated is needed to leverage this approach, and signal transduction pathways and effector genes mediating this phenotype are only beginning to be defined. Here, we show that calcineurin acts as a general switchboard that can both increase and decrease ß(1,3)-glucan exposure in response to multiple stimuli. Furthermore, we identify two novel downstream effector genes within the calcineurin regulon that drive this phenotype and impact virulence during systemic infection in mice.

## Introduction

In order to successfully disseminate, colonize and induce disease, fungal pathogens must overcome numerous stressors deployed by the host. These include limitations on nutrient availability [1–3], pH changes [4–6], or altered oxygen levels within specific host niches [7–10]. In addition to these barriers, a repertoire of pattern recognition receptors (PRRs) that recognize multiple components of the fungal cell wall are present on both hematopoietic and non-hematopoietic immune cells to effectively recognize and mount an immune response to the invading fungal pathogen. These include receptors that sense mannosylated proteins on the outermost cell wall, as well as PRRs for structural polysaccharides within the cell wall’s basal layers [11]. Amongst these basal layer polysaccharides, ß(1,3)-glucan is highly immunogenic, and PRRs that recognize exposed (unmasked) ß(1,3)-glucan moieties include dectin-1 [12–17], complement receptor 3 (CR3) [18,19] and the ephrin type A receptor 2 (EphA2) [20,21]. These receptors induce strong pro-inflammatory immune responses and are necessary for efficient fungal clearance. Thus, leveraging the impact that unmasked ß(1,3)-glucan has on host-pathogen interactions is a potential immunotherapeutic strategy for disease control.

In response to host derived environmental and immune-mediated stressors, fungal pathogens employ strategies to sense their surrounding environments and alter their levels of exposed ß(1,3)-glucan. *Aspergillus fumigatus* uses galactosaminogalactan or hydrophobins to cover (mask) ß(1,3)-glucan in hyphae and conidia, respectively [22,23]. Similarly, *Histoplasma capsulatum* masks ß(1,3)-glucan under a layer of α-glucan, and further reduces unmasked foci via expression of the glycosylhydrolyase Eng1 [24,25]. *Candida albicans* covers ß(1,3)-glucan in its cell wall under an outer layer of densely populated mannoproteins, and mutations that prevent proper glycosylphosphatidylinositol (GPI)-anchored protein synthesis or protein mannosylation induce unmasking [26–28]. Similarly, *C*. *albicans* senses environmental signals and stressors, such as lactate [29,30], acidic pH [31,32], hypoxia [33], and iron-replete environments [34] and responds by further altering ß(1,3)-glucan exposure. These stimulus driven events are mediated by activation of appropriate signal transduction pathways, such as the cyclic AMP/Protein Kinase A (cAMP/PKA) pathway [33,34] and the Cek1 mitogen activated protein kinase (MAPK) pathway [35–37]. These pathways therefore represent targets that can be utilized to better understand how ß-glucan exposure is regulated by *C*. *albicans*. Yet, our understanding of how these signaling pathways work together to regulate overall ß-glucan exposure is only beginning to be understood, and their downstream effector genes driving these structural changes remain to be fully elucidated.

Hyperactivation of the Cek1 MAPK pathway induces unmasking and attenuates virulence through the Cph1 transcription factor in a manner that is dependent, primarily, on a functional immune response [35–37]. The transcriptional regulon downstream of the Cek1 pathway includes the Dfi1 cell wall sensor, and Dfi1 mediates a significant portion of Cek1-induced ß(1,3)-glucan unmasking through a previously unidentified parallel signaling pathway [37]. Here, we expand on these findings and show that the calmodulin/calcineurin pathway is the parallel signaling pathway working downstream of Dfi1 and in synergy with the Cek1 MAPK pathway to induce the full levels of unmasking observed during Cek1 hyperactivation. Moreover, we show that calcineurin plays a more general role in mediating ß-glucan exposure, and is necessary to induce unmasking in response to the echinocandin caspofungin. In contrast, calcineurin reduces ß(1,3)-glucan exposure in response to extracellular calcium via the transcription factor Crz1. Using this information we identify two novel cell wall proteins, Fgr41 and Cwp419, that are necessary for maintaining homeostatic levels of ß(1,3)-glucan exposure, and show that loss of Fgr41 leads to reduced fungal burden in mice during systemic infection in a manner that is host immune system dependent.

## Results

### Ste11^ΔN467^-induced Dfi1 activation causes unmasking through the calmodulin-calcineurin signal transduction pathway

**Fig 1:**
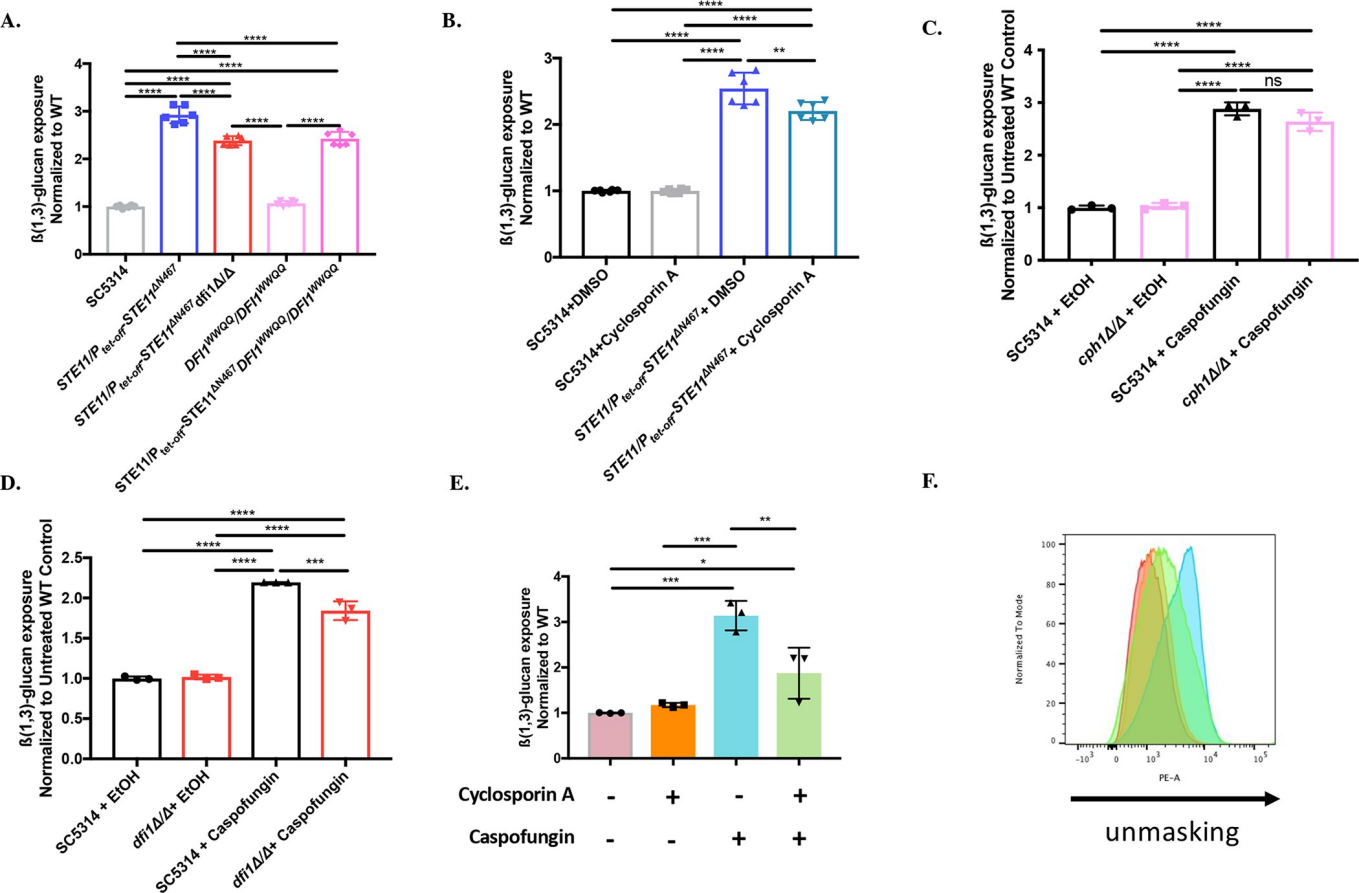
Calcineurin mediates hyperactive Ste11^*ΔN467*^-induced and caspofungin-induced unmasking in *C*. *albicans*. (A) Disruption of the Dfi1-calmodulin interaction reduces Ste11^*ΔN467*^-induced unmasking. Cells from overnight cultures were stained with an anti-ß(1,3)-glucan antibody and a phycoerythrin-conjugated secondary antibody, and flow cytometry analysis was used to assess the levels of ß(1,3)-glucan exposure. (n = 3 biological replicates with 1 technical replicate for each)(****p<0.0001, by one-way ANOVA). (B) Cyclosporine A inhibition of calcineurin reduces Ste11^*ΔN467*^-induced unmasking. The SC5314 wild-type or the *STE11/P*_*tet-off*_*-STE11*^*ΔN467*^ mutant was grown overnight in either the presence of 100μg/ml of cyclosporine A or an appropriate volume of the DMSO solvent control, and ß(1,3)-glucan exposure was assessed via flow cytometry. (n = 3 biological replicates with 1 technical replicate for each)(**p<0.005, ****p<0.0001, by one-way ANOVA). (C-D) Genes that are necessary for Ste11^*ΔN467*^-induced unmasking were tested for their roles in caspofungin-induced unmasking by treating cells grown to mid-log phase with 46.9ng/ml of caspofungin for 30 minutes followed by flow cytometry. (C) ß(1,3)-glucan unmasking in *cph1Δ/Δ* cells treated with sublethal concentrations of caspofungin. (n = 3 biological replicates)(****p<0.0001, by one-way ANOVA). (D) ß(1,3)-glucan unmasking in *dfi1Δ/Δ* cells treated with sublethal concentrations of caspofungin. (n = 3 biological replicates)(***p = 0.0005, ****p<0.0001, by one-way ANOVA). (E) Calcineurin inhibition attenuates caspofungin-induced unmasking. SC5314 wild-type cells grown to mid-log phase in either the presence of 100μg/ml of cyclosporine A, or an appropriate volume of the DMSO solvent control, were exposed to 46.9ng/ml of caspofungin or ethanol control for 30 minutes, and ß(1,3)-glucan exposure was measured via flow cytometry. (n = 3 biological replicates)(*p<0.05, **p<0.01, ***p<0.0005, by one-way ANOVA). (F) Representative histogram of the impact that cyclosporine A-mediated calcinuerin inhibition has on caspofungin induced ß(1,3)-glucan exposure. Colors of each sample match those shown in 1E.

We have previously shown that expression of a single hyperactive allele of the MAP3K *STE11* (*STE11*^*ΔN467*^) under the control of a tetracycline repressible promoter (*P*_*tet-off*_) induces unmasking through both the Cek1 MAPK pathway [35,36], and a previously unidentified pathway that is activated by the cell wall sensor Dfi1 [37]. Thus, in order to identify effector proteins driving Ste11^ΔN467^-induced unmasking, it was necessary to elucidate the parallel signaling pathway that contributes to the ß(1,3)-glucan exposure observed during *STE11*^*ΔN467*^ expression. Dfi1 has been shown to interact with calmodulin through a calmodulin binding motif in its C-terminal tail [38]. We hypothesized that, once expression is enhanced by *STE11*^*ΔN467*^, Dfi1 interacts with calmodulin to further increase ß(1,3)-glucan unmasking. To test this, we introduced two point mutations, W305Q and W308Q, into the C-terminal calmodulin binding motif of Dfi1. These mutations impair Dfi1-calmodulin interactions *in vitro* [38]. As previously reported [37], immunofluorescent staining with an anti-ß(1,3)-glucan antibody showed a significant increase in ß(1,3)-glucan exposure in the *STE11/P*_*tet-off*_*-STE11*^*ΔN467*^ mutant when compared to the wild-type (Figs 1A and S1). Moreover, deletion of *DFI1* in the *STE11/P*_*tet-off*_*-STE11*^*ΔN467*^ strain reduced its unmasking. Introduction of the W305Q and W308Q mutations in Dfi1 at residues critical for Dfi1-calmodulin binding reduced Ste11^ΔN467^-induced unmasking to the level observed in the *STE11/P*_*tet-off*_*-STE11*^*ΔN467*^*dfi1Δ/Δ* mutant. Thus, Dfi1 mediates Ste11^ΔN467^-induced unmasking through an interaction with calmodulin.

One of the functions of calmodulin is to activate calcineurin, which is a phosphatase that can propagate signal transduction that affects the cell wall [39–42]. Therefore, we assessed if signal transduction is mediated through calcineurin to induce the full levels of unmasking caused by *STE11*^*ΔN467*^ expression. Yeast cells were grown overnight in the presence of the calcineurin inhibitor cyclosporine A [43,44], and ß(1,3)-glucan unmasking was assessed by flow cytometry. Similarly to *STE11/P*_*tet-off*_*-STE11*^*ΔN467*^*DFI1*^W305Q,W308Q^/*DFI1*^W305Q,W308Q^ double mutant cells (Fig 1A), cyclosporine A treated cells exhibited a partial block of Ste11^ΔN467^-induced unmasking (Fig 1B). These data indicate that once stimulated by Dfi1, calmodulin mediates signal transduction through calcineurin to induce unmasking during *STE11*^*ΔN467*^ expression.

### The calcineurin pathway plays a role in mediating caspofungin-driven unmasking

Since unmasking by the Cek1 MAPK pathway is mediated in part by the calmodulin/calcineurin pathway [35–37] (Fig 1), we assessed if these pathways play a more general role in mediating unmasking induced by additional stimuli. Exposure to a sublethal concentration of the echinocandin caspofungin induces unmasking [45,46], and both the Cek1 and the calcineurin pathways are activated in response to caspofungin treatment [47–49]. Therefore, we hypothesized that these pathways help drive caspofungin-induced unmasking. To assess this, we tested mutants that block Ste11^ΔN467^-induced unmasking, including a *cph1Δ/Δ* mutant (transcription factor downstream of Cek1) [37] and the *dfi1Δ/Δ* mutant. Wild-type and mutant cells were grown to mid-log phase before being exposed to a sublethal concentration of caspofungin and ß(1,3)-glucan exposure was subsequently measured. Although Cph1 was the major transcription factor driving Ste11^ΔN467^-induced unmasking [37], deletion of *CPH1* did not significantly impact caspofungin-induced unmasking (Fig 1C). However, deletion of *DFI1* significantly reduced the unmasking caused by caspofungin treatment by ~16% (Fig 1D). This observation suggested that the calmodulin/calcineurin pathway, but not the Cek1 MAPK pathway, plays a role in driving caspofungin-induced unmasking. To directly test this hypothesis, we pharmacologically inhibited calcineurin with cyclosporine A and assessed the unmasking levels following caspofungin treatment. Inhibition of calcineurin with cyclosporine A caused a significant, ~41% reduction in the unmasking induced by caspofungin (Fig 1E & 1F). Therefore, calcineurin plays a role in mediating unmasking during both hyperactive *STE11*^*ΔN467*^ expression and in response to sublethal concentrations of caspofungin.

### Growth in exogenous calcium induces masking in *C*. *albicans*

**Fig 2:**
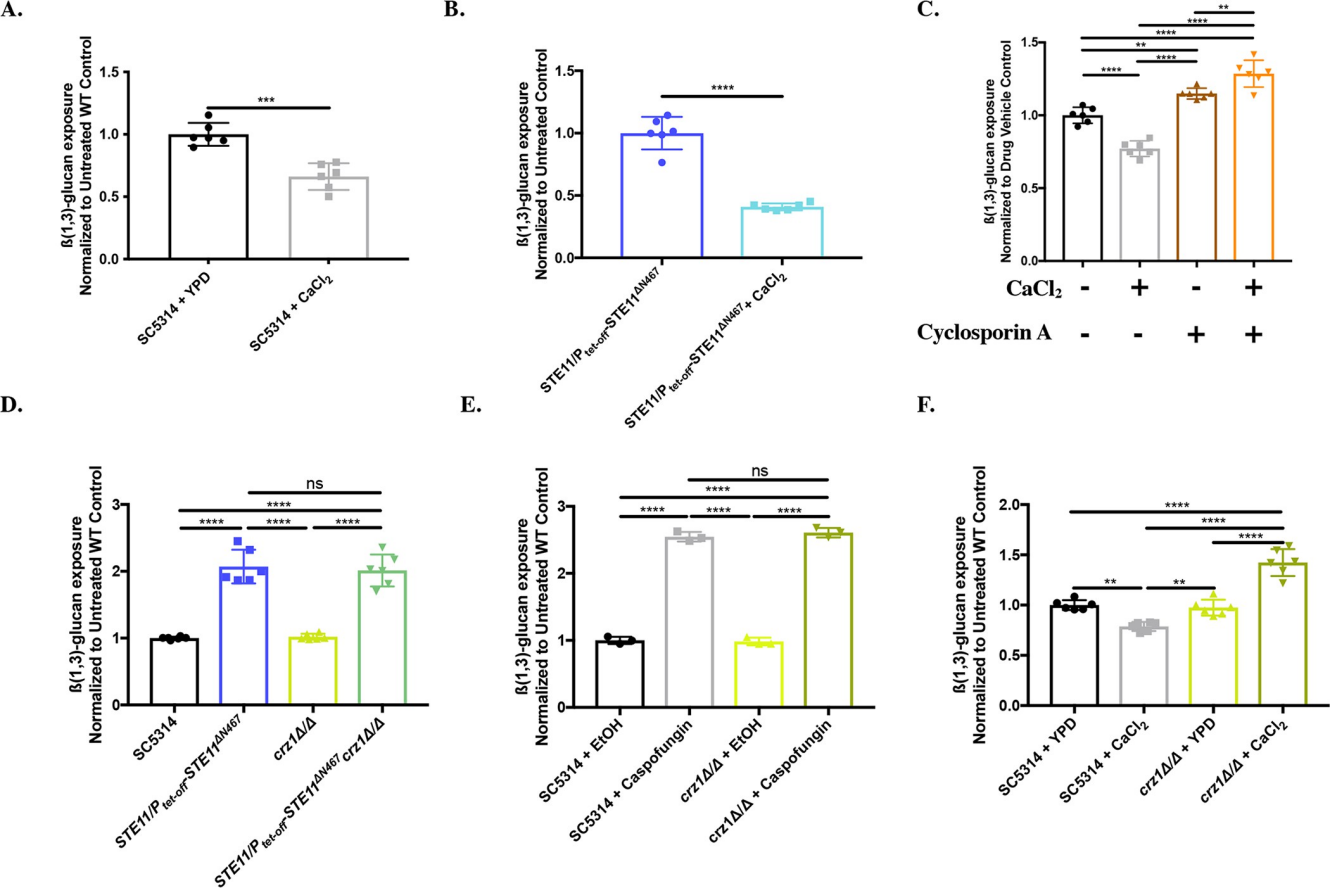
Crz1 mediates masking in response to exogenous calcium. (A-B) Cells were grown overnight in the absence or presence of 50mM CaCl_2_ in YPD broth. Cells were stained with an anti-ß(1,3)-glucan antibody and a phycoerythrin-conjugated secondary antibody followed by flow cytometry to assess the levels of ß(1,3)-glucan exposure. (A) Calcium-induced masking of SC5314 wild-type cells. (n = 3 biological replicates with 1 technical replicate for each) (***p = 0.0002, by student’s t-test). (B) Calcium induced masking of *STE11/P*_*tet-off*_*-STE11*^*ΔN467*^ cells. (n = 3 biological replicates with 1 technical replicate for each) (****p<0.0001, by student’s t-test). (C) Calcineurin inhibition blocks calcium-induced masking. SC5314 cells were grown overnight in the absence or presence of 50mM CaCl_2_ and either 100μg/ml of cyclosporine A or an appropriate volume of the DMSO solvent control in YPD media. ß(1,3)-glucan exposure was then assessed via flow cytometry. (n = 3 biological replicates with 1 technical replicate for each)(**p<0.01, ****p<0.0001, by one-way ANOVA). (D) Loss of *CRZ1* does not impact Ste11^*ΔN467*^-induced unmasking. Overnight cultures were stained for ß(1,3)-glucan exposure and flow cytometry was subsequently run to assess unmasking. (n = 3 biological replicates with 1 technical replicate for each) (ns = not significant, ****p<0.0001, by one-way ANOVA). (E) Loss of *CRZ1* does not impact caspofungin-induced unmasking. Cells grown to mid-log phase prior to being exposed to 46.9ng/ml of caspofungin for 30 minutes and ß(1,3)-glucan exposure was subsequently assessed via flow cytometry. (n = 3 biological replicates)(ns = not significant, ****p<0.0001, by one-way ANOVA). (F) Loss of *CRZ1* blocks calcium-induced masking. Overnight cultures of cells grown in the absence or presence of 50mM CaCl_2_ were stained for ß(1,3)-glucan exposure and flow cytometry was subsequently run to assess ß(1,3)-glucan exposure levels. (n = 3 biological replicates with 1 technical replicate for each)(**p<0.01, ****p<0.0001, by one-way ANOVA).

The observation that calcineurin induces unmasking during both hyperactive *STE11*^*ΔN467*^ expression and in response to caspofungin (Fig 1) raised the possibility that the calcineurin pathway may induce unmasking upon direct stimulation. The calmodulin/calcineurin pathway mediates signaling in response to high levels of exogenous calcium [50,51]. We hypothesized that activation of the calcineurin pathway via calcium addition would also increase ß(1,3)-glucan exposure. Thus, cells were grown in the presence of 50mM CaCl_2_, but unexpectedly, these cells had significantly less ß(1,3)-glucan exposure (an ~34% reduction) than wild-type cells grown in non-supplemented media (Fig 2A). This trend was even more apparent when the already unmasked *STE11/P*_*tet-off*_*-STE11*^*ΔN467*^ mutant was grown in media with 50mM calcium. The extracellular calcium significantly reduced Ste11^ΔN467^-induced unmasking to only ~40% of the levels observed during growth in YPD alone (Fig 2B). Interestingly, calcineurin inhibition via cyclosporine A treatment blocked the masking induced by exogenous calcium in wild-type cells, and actually resulted in increased unmasking (Fig 2C). Therefore, growth in high levels of extracellular calcium can induce masking in *C*. *albicans* in a calcineurin dependent manner.

### Crz1 mediates calcineurin induced masking, but not unmasking

Our results have shown that calcineurin plays a role in regulating ß(1,3)-glucan exposure in response to specific stimuli. However, calcineurin’s impacts on epitope exposure appear to be stimulus-dependent, with caspofungin treatment and *STE11*^*ΔN467*^ expression both increasing ß(1,3)-glucan exposure in a calcineurin-dependent manner (Fig 1), and exposure to high levels of exogenous calcium inducing masking via calcineurin (Fig 2A–2C). To address these differences, we tested the impact that the downstream transcription factor Crz1 has on regulating these changes. Crz1 is the canonical transcription factor that sits downstream of calcineurin in the calcium response pathway [52–54]. Its activation has also been shown to induce masking in response to exogenous lactate, although this occurs in a manner that is independent of calcineurin [29,30]. Due to the masking induced by activation of the canonical Ca^2+^/Calmodulin/Calcineurin pathway, we hypothesized that Crz1 mediates masking during exposure to high levels of extracellular calcium, but is likely not involved in the unmasking induced by non-canonical calcineurin activation in response to *STE11*^*ΔN467*^ expression or caspofungin treatment. To test this, we generated a *crz1Δ/Δ* mutation in both the wild-type and *STE11/P*_*tet-off*_*-STE11*^*ΔN467*^ parent strains and tested the impact that loss of this transcription factor had on regulating ß-glucan exposure. Loss of *CRZ1* did not impact unmasking during *STE11*^*ΔN467*^ expression (Fig 2D), nor did it have an impact on caspofungin-induced unmasking (Fig 2E). However, the *crz1Δ/Δ* mutant failed to induce masking in response to exogenous calcium (Fig 2F), and actually resulted in an increase in unmasking compared to wild-type cells grown in non-supplemented YPD. This is consistent with the impact that chemical inhibition of calcineurin had on calcium-induced masking (Fig 2C) and provides strong support for Crz1’s role in specifically mediating epitope masking in response to high extracellular calcium levels.

### Loss of key cell wall proteins causes ß(1,3)-glucan unmasking

**Fig 3:**
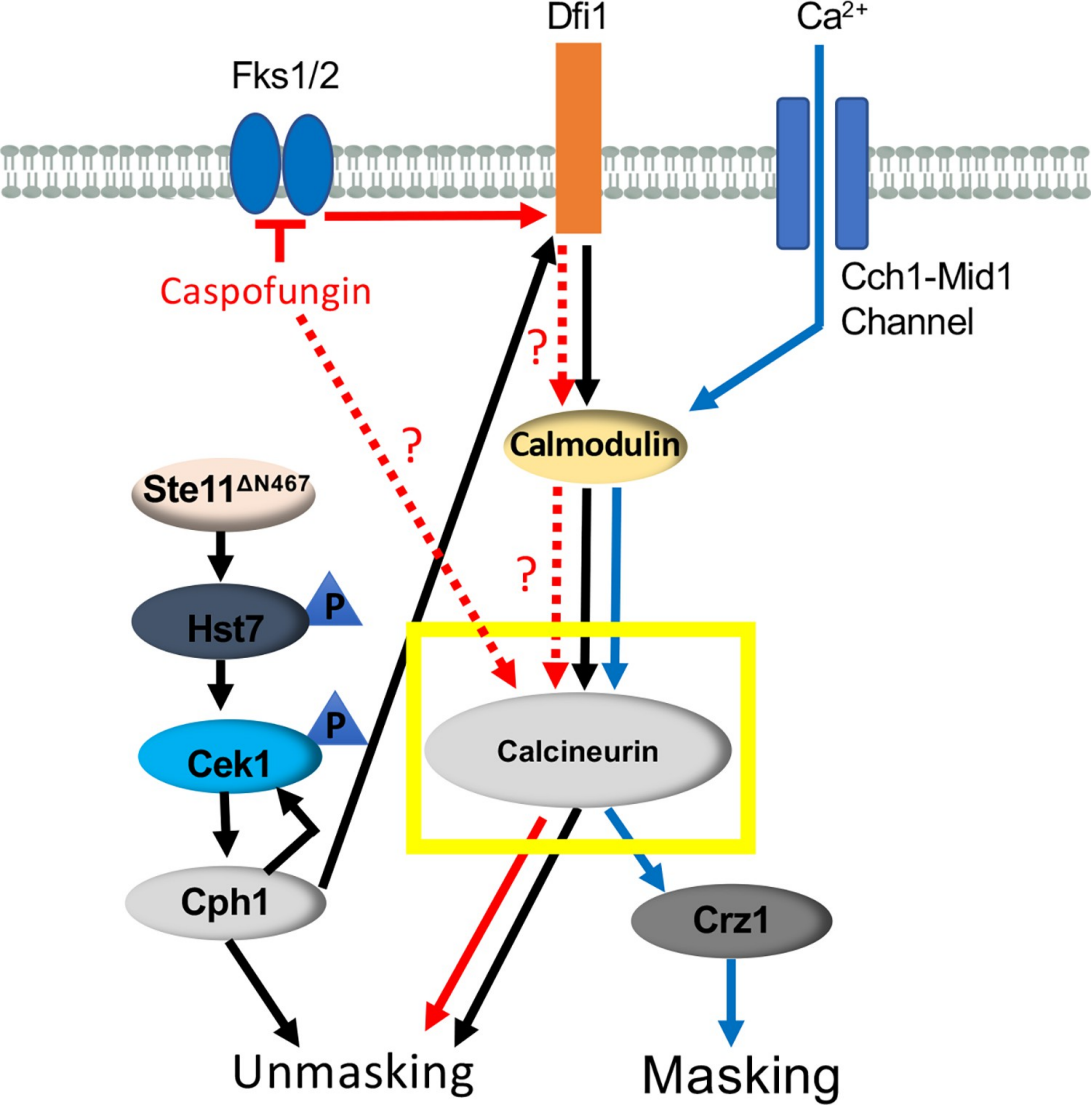
Proposed model for calcineurin-regulated ß(1,3)-glucan exposure. Our data suggest that calcineurin serves as a switchboard in mediating ß(1,3)-glucan exposure in response to different stimuli: hyperactive *STE11*^*Δ*N467^ expression (black lines), caspofungin exposure (red lines), and high levels of exogenous calcium (blue lines). With regards to hyperactive *STE11*^*Δ*N467^ expression, we propose that activation of the downstream transcription factor Cph1 leads to increased expression of the cell wall sensor Dfi1 as well as architectural cell wall changes that are then sensed by these same Dfi1 proteins. This sensor in turn interacts with calmodulin to initiate signal transduction down a Dfi1/calmodulin/calcineurin signal transduction pathway and further increases ß(1,3)-glucan unmasking. In response to caspofungin exposure, calcineurin is also necessary to induce the full levels of unmasking observed in wild-type cells. Based on our data, calcineurin may act in a partially Dfi1 dependent manner, but an alternative regulator also mediates calcineurin activity in response to caspofungin treatment. Lastly, we propose that calcineurin is also capable of decreasing ß(1,3)-glucan exposure levels in response to growth in high levels of exogenous calcium, and is likely mediated via the canonical calmodulin/calcineurin/Crz1 signal transduction pathway. Dotted arrows indicate putative pathways that have not yet been experimentally confirmed.

**Fig 4:**
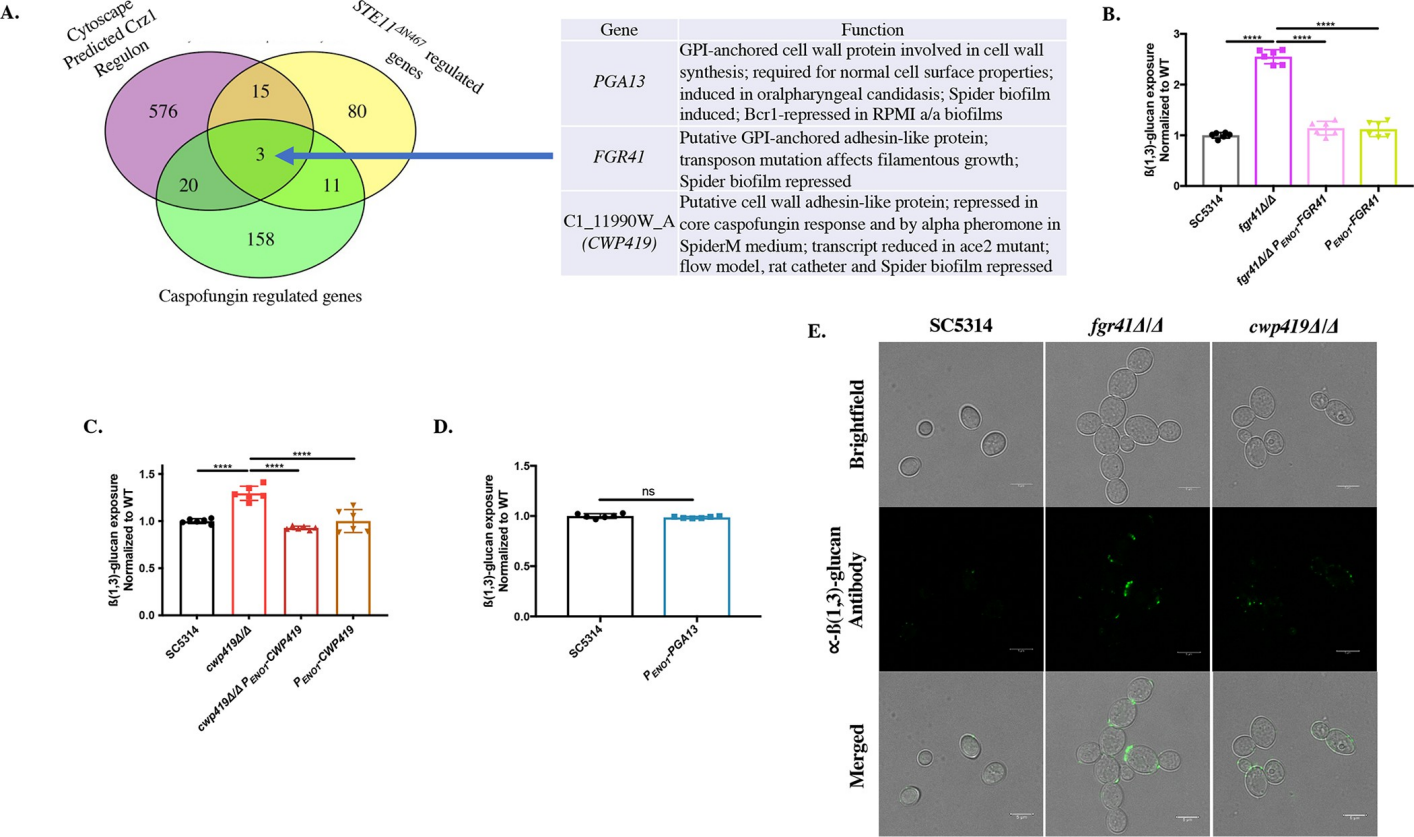
Key cell wall proteins mediate ß(1,3)-glucan exposure in *C*. *albicans*. (A) Comparative analysis of public data sets to identify conserved genes that are differentially regulated in conditions that involve calcineurin-mediated ß(1,3)-glucan exposure (*STE11*^*Δ*N467^ expression [36] and caspofungin exposure [55]) and fall within a predicted Crz1 regulon [29]. Gene ontology descriptions were taken from the Candida Genome Database. (B-D) Genetic analysis of conserved target genes identified during comparative analysis. Overnight cultures of cells were stained with anti-ß(1,3)-glucan antibody and a phycoerythrin-conjugated secondary antibody followed by flow cytometry analysis to assess the levels of ß(1,3)-glucan exposure. (B) ß(1,3)-glucan unmasking of *fgr41Δ/Δ* mutants. (C) ß(1,3)-glucan unmasking of *cwp419Δ/Δ* mutants. (D) ß(1,3)-glucan unmasking of *PGA13* overexpression mutants. (n = 3 biological replicates with 1 technical replicate for each mutant analyzed)(****p<0.0001, by one-way ANOVA). (E) Representative microscopy images of ß(1,3)-glucan exposure of the *fgr41Δ/Δ* and *cwp419Δ/Δ* mutants. (The scale bar indicates 5μm).

Our data have thus far shown a role for calcineurin in regulating ß(1,3)-glucan exposure in response to three distinct stimuli: *STE11*^*ΔN467*^ expression, caspofungin exposure and growth in high extracellular concentrations of calcium (Figs 1 and 2). We have also shown that masking in response to exogenous calcium is induced by the transcription factor Crz1, which has been additionally implicated in regulating masking in response to extracellular lactate [29,30]. This creates a model in which calcineurin serves as a node within multiple signaling pathways to regulate ß(1,3)-glucan exposure in *C*. *albicans* (Fig 3). However, *STE11*^*ΔN467*^ expression, caspofungin exposure and exogenous calcium all cause large changes in the cell wall architecture and metabolism of *C*. *albicans* [36,37,54–56]. Calcineurin activation and subsequent ß(1,3)-glucan (un)masking are one set of multiple changes induced by these stimuli, and effector genes that impact ß(1,3)-glucan exposure are likely a small subset of the total genes differentially regulated in the *C*. *albicans* transcriptional response to each stimulus. Identifying shared genes that are induced in response to all of these stimuli may be an efficient way to highlight conserved effector genes within the calcineurin regulon that drive changes in ß(1,3)-glucan exposure in response to multiple stimuli. Consequently, we utilized public data sets to identify genes that are differently regulated during both *STE11*^*ΔN467*^ expression [36] and caspofungin treatment [55], and overlaid them with a Cytoscape predicted Crz1 regulon [29]. Overlapping these datasets revealed that only 3 genes were conserved amongst them (Fig 4A); *PGA13*, *FGR41* and a gene of unknown function *C1_11990W_A* (which we have termed cell wall protein 419, *CWP419)*. Interestingly, all three genes are predicted to encode outer cell wall proteins. All three contain putative secretion signals and two of the three (*PGA13* and *FGR41*) have predicted glycosylphosphatidylinositol anchors in their sequences. This observation fits the model that alterations in outer cell wall components are capable of affecting ß(1,3)-glucan exposure [26–28] and encouraged further exploration of these targets.

Within the datasets used to identify the effector genes, *FGR41* and *CWP419* were both down-regulated during *STE11*^*ΔN467*^ expression and caspofungin exposure, while *PGA13* was up-regulated in each condition [36,55]. In contrast, *FGR41* and *CWP419* are predicted to be positively regulated by Crz1, while *PGA13* is predicted to be down-regulated [29]. Thus, the differential expression profiles of these genes in conditions that either increase or decrease ß(1,3)-glucan exposure provides a basis to assess their contributions to unmasking. To recapitulate the role that these genes may have in mediating unmasking in response to these stimuli, we created knockout mutants of *FGR41* and *CWP419*, and an overexpression mutant of *PGA13* under the control of the enolase promoter (*P*_*ENO1*_*-PGA13*). Interestingly, both *fgr41Δ/Δ* (Fig 4B) and *cwp419Δ/Δ* (Fig 4C) mutants showed a significant increase in unmasking compared to their respective wild-type and reintegrant controls, while overexpression of *PGA13* appeared to have no impact on ß-glucan exposure (Fig 4D). Microscopy further revealed that unmasking in the *fgr41Δ/Δ* mutant occurs primarily at the bud neck and bud scars, while the *cwp419Δ/Δ* mutant showed a more scattered pattern of unmasking throughout the periphery of the cell wall (Fig 4E). Thus, it appears that down-regulation of *FGR41* and *CWP419* may be responsible, in part, for the unmasking observed during *STE11*^*ΔN467*^ expression and caspofungin exposure.

As loss of either *FGR41* or *CWP419* increased unmasking, we were curious if changes in other structural components of the cell wall were also present in either of these mutants. To assess this, the wild-type, *fgr41Δ/Δ* and *cwp419Δ/Δ* strains were stained with calcofluor white, wheat germ agglutinin and concanavalin A to assess the levels of total chitin, exposed chitin and mannan within their cell walls, respectively. Staining revealed that the *fgr41Δ/Δ* mutant displayed significant increases in both total chitin and mannan within its cell wall, while the *cwp419Δ/Δ* mutant displayed wild-type levels of staining for these cell wall components (S2 Fig). Furthermore, both mutants displayed a very modest, but significant increase in exposed chitin levels as well, accounting for only a ~1.10 to 1.20-fold increase in wheat germ agglutinin staining. Although we suspect that the increase in exposed chitin is not biologically significant within the samples, our data does suggest that loss of *FGR41*, but not *CWP419*, induces robust changes in the cell wall architecture of *C*. *albicans*.

**Fig 5:**
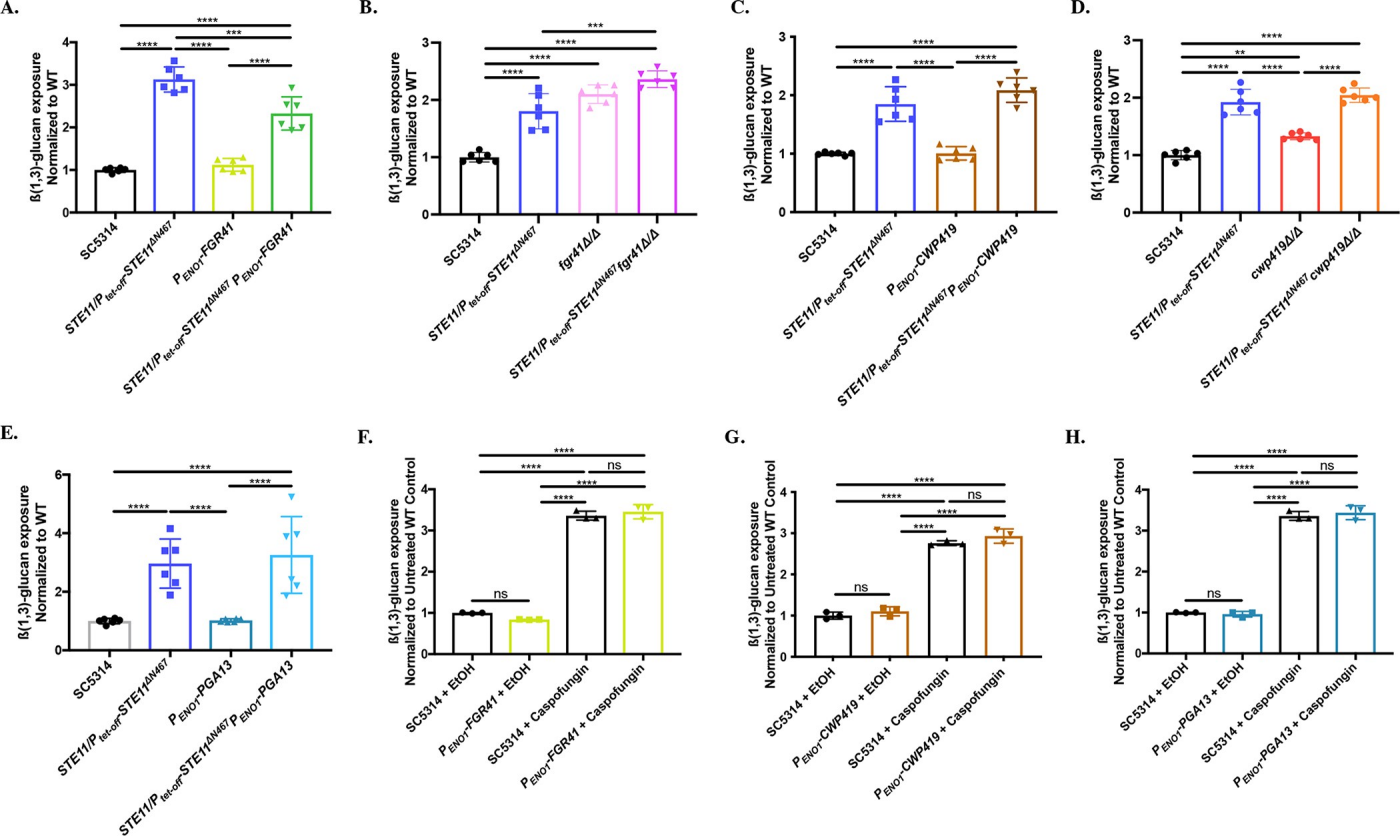
*FGR41* overexpression and deletion modulates Ste11^ΔN467^-induced, but not caspofungin-induced, ß(1,3)-glucan unmasking. (A-E) Overnight cultures of cells were stained with anti-ß(1,3)-glucan antibody and a phycoerythrin-conjugated secondary antibody to assess the levels of ß(1,3)-glucan exposure by flow cytometry. (A) ß(1,3)-glucan exposure during *FGR41* overexpression. (B) ß(1,3)-glucan exposure in *FGR41* deletion mutants. (C) ß(1,3)-glucan exposure during *CWP419* overexpression. (D) ß(1,3)-glucan exposure in *CWP419* deletion mutants (E) ß(1,3)-glucan exposure during *PGA13* overexpression. (n = 3 biological replicates with 1 technical replicate for each mutant analyzed)(**p<0.005, ***p<0.0005, ****p<0.0001, by one-way ANOVA). (F-H) Cells grown to mid-log phase were exposed to 46.9ng/ml of caspofungin for 30 minutes and ß(1,3)-glucan exposure was subsequently assessed via flow cytometry. (F) ß(1,3)-glucan exposure of *FGR41* overexpression cells treated with sublethal concentrations of caspofungin. (G) ß(1,3)-glucan exposure of *CWP419* overexpression cells treated with sublethal concentrations of caspofungin. (H) ß(1,3)-glucan exposure of *PGA13* overexpression cells treated with sublethal concentrations of caspofungin. (n = 3 biological replicates)(ns = not significant, ****p<0.0001, by one-way ANOVA).

We next wished to see if differential gene regulation of any of these constructs could impact the unmasking induced by *STE11*^*ΔN467*^ expression or caspofungin exposure. Overexpression of *FGR41* was able to reduce the unmasking observed during *STE11*^*ΔN467*^ expression (Fig 5A). In addition, deletion of *FGR41* in a *STE11/P*_*tet-off*_*-STE11*^*ΔN467*^ background led to a further increase in ß-glucan exposure (Fig 5B). However, neither overexpression nor deletion of *CWP419*, nor overexpression of *PGA13* impacted Ste11^ΔN467^-induced unmasking (Fig 5C–5E). In contrast, none of the overexpression mutants were able to affect caspofungin-induced unmasking (Fig 5F–5H), which represents a much more pleiotropic impact on the cell wall.

Both *FGR41* and *CWP419* are predicted to be positively regulated by Crz1 [29], and the inability for the *crz1Δ/Δ* mutant to show calcium-induced masking may be the consequence of misregulation of these downstream targets. We were therefore curious if overexpression of each of these components alone could restore the ability of the *crz1Δ/Δ* mutant to induce masking in response to growth in exogenous calcium. However, in the presence of exogenous calcium, neither the *crz1Δ/ΔP*_*ENO1*_*-FGR41* nor the *crz1Δ/ΔP*_*ENO1*_*-CWP419* double mutant had altered ß(1,3)-glucan exposure when compared to the *crz1Δ/Δ* parent strain (S3 Fig). Therefore, it appears that overexpression of each of these targets alone is insufficient to restore calcium-induced masking in the *crz1Δ/Δ* mutant.

### Loss of *FGR41* attenuates virulence during systemic infection in mice

**Fig 6:**
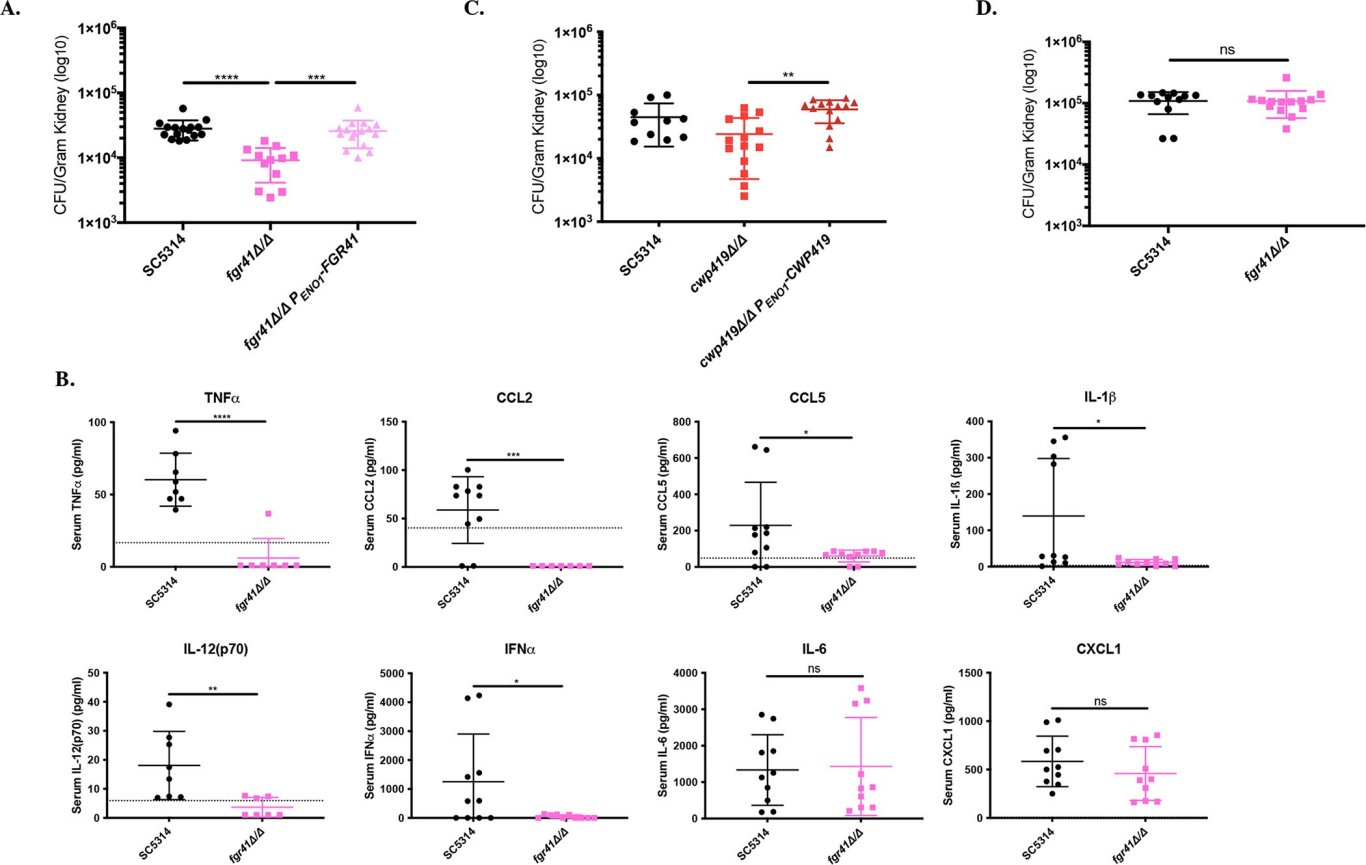
Loss of *FGR41* attenuates virulence in a host immune system dependent manner. (A) ICR mice were intravenously infected with 1x10^6^ cells of *C*. *albicans* wild-type (SC5314), the *fgr41Δ/Δ* mutant, or the *fgr41Δ/ΔP*_*ENO1*_*-FGR41* reintegrant strain, and kidneys were subsequently harvested 4 days post infection (d.p.i.) to assess fungal burden. (n = 7–8 mice per strain)(***p<0.0005, ****p<0.0001, by Kruskal-Wallis test with Dunn’s multiple comparisons post-hoc analysis). (B) Serum collected 4 d.p.i. was assessed via flow cytometry using the LEGENDplex cytokine bead-based array kit to measure circulatory cytokine levels following infection with either the *C*. *albicans* wild-type (SC5314) or the *fgr41Δ/Δ* mutant. (n = 5 mice)(The dashed line across the x-axis represents the limit of detection.)(ns = not significant, *p<0.05, **p<0.01, ***p = 0.0005, ****p<0.0001, by either student’s t-test or Mann-Whitney test). (C) ICR mice were intravenously infected with 1x10^6^ cells of *C*. *albicans* wild-type (SC5314), the *cwp419Δ/Δ* mutant, or the *cwp419Δ/ΔP*_*ENO1*_*-CWP419* reintegrant strain, and kidneys were subsequently harvested 4 d.p.i. to assess fungal burden. (n = 6–8 mice per strain)(**p<0.01, by one-way ANOVA). (D) ICR mice were immunosuppressed with recurring intraperitoneal injections of 150mg/kg of cyclophosphamide every 3 days starting 4 days prior to infection. At day 0, mice were intravenously injected with 1x10^4^ cells of *C*. *albicans* wild-type (SC5314) or the *fgr41Δ/Δ* mutant and kidneys were harvested 3 days post infection (d.p.i.) to assess fungal burden. (n = 6–7 mice)(ns = not significant, by Mann-Whitney test).

As both *fgr41Δ/Δ* and *cwp419Δ/Δ* display increased unmasking, and neither displays an aberrant growth rate *in vitro* (S4 Fig), we measured the impact that these mutations had on systemic infection in mice. We intravenously infected mice with 1x10^6^ cells of wild-type, *fgr41Δ/Δ*, *P*_*ENO1*_*-FGR41*, *cwp419Δ/Δ*, or their respective reintegrants and determined fungal burden and serum cytokine levels 4 days post infection (d.p.i). Mice infected with the *fgr41Δ/Δ* mutant displayed a significant (~3-fold) reduction in kidney fungal burden at 4 d.p.i. (Fig 6A), with an accompanying decrease in core pro-inflammatory cytokines, like TNFα, CCL2, CCL5, IL-1ß and IFNα (Figs 6B and S5). However, the *P*_*ENO1*_*-FGR41* mutant displayed wild-type levels of kidney fungal burden (S6 Fig). The *cwp419Δ/Δ* mutant displayed a modest and insignificant decrease in fungal burden compared to the wild-type, but was significantly different than the reintegrant (Fig 6C), and this is likely reflective of the modest increase in unmasking induced by loss of this gene (Fig 4C).

*FGR41* is a putative GPI-anchored protein, and transposon library screening has implicated it as a regulator for filamentous growth [57]. Since hyphal formation is an important virulence factor, we tested the impact of *fgr41Δ/Δ* on the yeast-to-hyphae transition *in vitro*. The *fgr41Δ/Δ* showed a significant reduction in the percentage of cells that formed germ tubes in the first 2 hours post-induction in serum, but by 3 hours there was no significant difference (S7A Fig). Additionally, of hyphae that were generated at 1 or 2 hours, there was a significant increase in *fgr41Δ/Δ* hyphal length, but by 3 hours there was no significant difference (S7B Fig). Thus, there was only a modest impact on early hyphal formation in *the fgr41*Δ/Δ mutant.

The ability to adapt to exogenous stressors that impact the integrity of the cellular envelope is an additional variable that may contribute to observed virulence defects seen *in vivo*. However, neither the *fgr41Δ/Δ* mutant nor the *cwp419Δ/Δ* mutant displayed altered sensitivities to calcofluor white, caspofungin or sodium dodecyl sulfate (SDS) during spot dilution assays (S8 Fig). Therefore, no apparent fitness defects appear to exist to these common cell wall stressors that may be indicative of reduced cell wall integrity in these mutants.

To more clearly assess if the unmasking phenotype of the *fgr41Δ/Δ* mutant was responsible for the reduced fungal burden, we determined if the virulence defect was dependent on the host immune response. We tested this by repeating the infection with 1x10^4^ cells in cyclophosphamide immunosuppressed mice and analyzing kidney fungal burden at 3 d.p.i. (to prevent mice from pre-maturely succumbing to the infection). Cyclophosphamide treatment depleted cell types from both the adaptive and innate immune response (S9 Fig) and completely restored *fgr41Δ/Δ* fungal burden to wild-type levels (Fig 6D). Thus, the reduced fungal burden during *fgr41Δ/Δ* infection is dependent on an intact host immune system. This suggests that unmasking of ß(1,3)-glucan plays the driving role in virulence attenuation, rather than an overall fitness defect or filamentation delay.

## Discussion

We have previously shown that hyperactivation of the Cek1 MAPK pathway of *C*. *albicans* is capable of inducing ß(1,3)-glucan unmasking [35,36]. We have also shown that this is mediated by the transcription factor Cph1, and that its activation stimulates a parallel signaling pathway to further increase ß-glucan exposure [37]. Here we have expanded on this work to show that the calmodulin/calcineurin signaling pathway is this parallel pathway and is required to fully induce unmasking caused by *STE11*^*ΔN467*^ expression (Fig 1A and 1B). Furthermore, we have shown that calcineurin plays a more general role in mediating ß(1,3)-glucan exposure, as it is necessary for caspofungin-induced unmasking (Fig 1E and 1F). In contrast, we also show that calcineurin reduces ß(1,3)-glucan exposure during growth in media containing high levels of extracellular calcium (Fig 2). Thus, for caspofungin and Ste11^ΔN467^-induced unmasking, as well as calcium-induced masking, changes in ß-glucan exposure are mediated via the same calcineurin signal transduction module, but the mechanism of calcineurin activation differs between them (Fig 3).

The manner by which calcineurin is activated in response to sublethal concentrations of caspofungin is unknown. We have shown that deletion of *DFI1* impacts caspofungin-induced unmasking (Fig 1D), and it is possible that calcineurin is activated by caspofungin in part through the same Dfi1/calmodulin/calcineurin signal cascade stimulated by *STE11*^*ΔN467*^ expression. However, loss of calcineurin has a greater impact on caspofungin-driven unmasking than loss of Dfi1, so calcineurin may be induced by other inputs in addition to Dfi1. Sublethal concentrations of caspofungin can induce an apoptotic response in *C*. *albicans* [58,59], and are regulated by the interaction between the molecular chaperone Hsp90 and calcineurin [60,61]. Moreover, the calcium response pathway plays a role in mediating *C*. *albicans* echinocandin resistance [62], and Hsp90-mediated activation of calcineurin is essential for this process [49]. Therefore, Hsp90 may play a role in mediating calcineurin-dependent ß(1,3)-glucan unmasking in response to caspofungin. In any case, the observation that calcineurin is needed to induce the full levels of unmasking observed during caspofungin treatment is interesting, as the mechanism driving caspofungin-induced unmasking has not been previously described.

Caspofungin-driven unmasking may also play an important role in enchinocandin mechanism of action during infection, as there is evidence to suggest that a functional immune response is necessary for proper fungal clearance during echinocandin treatment in mice. Marakalala *et al*. reported that caspofungin efficacy is severely impaired during infection in dectin1^-/-^ mice, suggesting an essential role for this ß(1,3)-glucan receptor in mediating caspofungin-induced fungal clearance [63]. Similarly, the ability of anidulafungin to enhance host survival during systemic infection is impaired in neutropenic mice [64]. Thus, the impact that calcineurin has in regulating ß(1,3)-glucan exposure in response to caspofungin treatment may have additional implications on disease management via echinocandin application.

The role for calcineurin in regulating ß-glucan exposure levels in response to multiple stimuli is not surprising, as this protein complex is capable of regulating multiple phenotypic outcomes in response to pH [65,66], ion stress [42,65,67], antifungal tolerance [49,67] and serum growth [50,65,68]. Each of these processes requires tailored responses for *C*. *albicans* to adequately adapt. With respect to altering ß(1,3)-glucan exposure, we have found that this is in part mediated via directed activation of appropriate downstream transcription factors. In this report, Crz1 was found to specifically mediate calcium-induced masking but had no impact in unmasking conditions (Fig 2D–2F). The transcription factor acting downstream of calcineurin to control unmasking is currently unknown. The observation that Crz1 is capable of inducing masking is consistent with previous literature, as Crz1 has also been implicated in lactate-induced masking [29,30], although the manner in which it was activated in response to lactate exposure was non-canonical. Although the cAMP/PKA pathway is also capable of responding to multiple host signals to induce masking (lactate and hypoxia exposure, and iron-replete environments) [33,34], calcineurin is the first signal transduction pathway to our knowledge that can both increase and decrease ß-glucan exposure in response to different stimuli. As such, the role that the calmodulin/calcineurin pathway has on mediating ß-glucan exposure levels may serve as a model to better understand the checks and balances in place to maintain appropriate ß-glucan exposure in *C*. albicans, and to also begin addressing larger questions in eukaryotic cell biology. Namely, how signal transduction is regulated to activate alternative downstream transcription factors.

In an attempt to elucidate the mechanism driving calcineurin-induced changes in ß(1,3)-glucan exposure, we have identified two putative cell wall proteins, Fgr41 and Cwp419, that are down-regulated during *STE11*^*ΔN467*^ expression [36] and caspofungin exposure [55], and that cause unmasking when individually deleted (Fig 4). In agreement with this, these proteins are also predicted to be positively regulated by Crz1, which induces masking, and their differential expression may therefore contribute to the different calcineurin-mediated changes on ß(1,3)-glucan exposure. The observation that overexpression of *FGR41* can partially restore the unmasking induced by hyperactive *STE11*^*ΔN467*^ expression supports this model (Fig 5A). However, neither *FGR41* nor *CWP419* overexpression had an impact on caspofungin-induced unmasking (Fig 5F–5G). Additionally, overexpression of these genes was not able to overcome the loss of *CRZ1* to restore the masking phenotype observed during growth in exogenous calcium (S3 Fig). Yet, the transcriptional responses to both caspofungin and calcium exposure are profound, and we suspect that multiple variables contribute to induce the changes in ß(1,3)-glucan exposure observed. Indeed, in addition to differential regulation of outer cell wall proteins, caspofungin treatment also induces a significant increase in chitin content within the cell wall [69,70]. Calcineurin has been found to play a role in regulating chitin levels in response to caspofungin [69], and correlations with increased chitin content and increased unmasking have been repeatedly observed in the literature [37,45,71–73]. It is therefore possible that increased chitin levels may work in synergy with down-regulation of outer cell wall proteins, such as *FGR41* and *CWP419*, to induce unmasking.

Both Fgr41 and Cwp419 are largely uncharacterized proteins, and the direct role that they may play in cell wall homeostasis and ß(1,3)-glucan exposure are unknown. Loss of *FGR41* was previously shown to impair filamentation in *C*. *albicans* [57], and in our hands *fgr41Δ/Δ* only causes a delay in filamentous growth in a small percentage of cells (S7 Fig). Similarly, *CWP419* was implicated as a putative adhesin-like protein in the Ace2 regulon [74], but its biological role in *C*. *albicans* is thus far unreported. Knockout mutants of either gene did not show altered susceptibility to common cell envelope stressors (S8 Fig), and bioinformatic analysis using BLAST [75] and PROSITE [76] did not reveal any conserved protein domains to hint at their biological function. However, ß(1,3)-glucan staining did reveal that the *fgr41Δ/Δ* mutant had increased unmasking at bud scars and bud necks, and these cells appeared to have impaired septum resolution in microscopy (Fig 4E). Therefore, it may be that Fgr41 plays a role in cell division. The *cwp419Δ/Δ* mutant showed scattered foci of unmasking, and this may suggest a broader distribution of this protein throughout the cell wall. It is important to note that although other cell wall proteins have been found to induce unmasking when deleted, such the yeast wall protein *YWP1* [77], this phenotype is not conserved amongst all cell wall proteins. For example, we have shown that deletions of the cell wall sensors *DFI1* and *OPY2*, which intercalate into the outer cell wall, do not induce unmasking, even when both are simultaneously deleted [37]. Therefore, the uncharacterized nature of these proteins warrants further investigation to elucidate how they regulate cell wall homeostasis and ß-glucan exposure.

In the case of *FGR41*, we found that loss of this cell wall protein leads to virulence attenuation during systemic infection in mice in a manner that is dependent on the host immune system (Fig 6). This suggests that the virulence defect is dependent on unmasking rather than the modest filamentation delay. Although the mechanism by which Fgr41 impacts masking is unknown, this mutant should serve as a useful model to understand how unmasking is mediated by cell wall proteins. Furthermore, its application can also be used to assess the impact that unmasking has on immune-driven fungal clearance, without complications from strong *in vivo* fitness defects.

## Methods

### Growth media and culture conditions

*C*. *albicans* strains were grown in YPD media (1% yeast extract, 2% peptone, 2% dextrose)(Thermo Fisher Scientific) while shaking at 225rpm at 30°C [78]. In conditions where exogenous calcium was added, YPD broth was supplemented with CaCl_2_ to 50mM media [67]. Minimal media (0.67% yeast nitrogen base without amino acids, 2% dextrose, 2% agar) [78] (Thermo Fisher Scientific) was used to remove the genomically integrated CRISPR/Cas9 cassette and nourseothricin selectable marker following successful gene deletion [79]. LB media (0.5% yeast extract, 1% tryptone, 1% NaCl) (Thermo Fisher Scientific) was used for the growth of DH5-α *Escherichia coli* strains (NEB) and cells were grown at 37°C on a rotator drum.

### Plasmid construction

Plasmids constructed during this project can be seen in S1 Table and the primers used to create them in S2 Table. Overexpression cassettes were created by placing the open reading frame (ORF) for the gene of interest under the regulatory control of the constitutive enolase *(ENO1*) promoter [80]. To create the *FGR41* overexpression cassette, a 1,023bp fragment containing the *FGR41* open reading frame (ORF) and 270bp of its 3’ untranslated region (UTR) following the stop codon was amplified with the AWO361 and AWO362 primer set that introduced flanking *NotI* cut sites onto both ends of the PCR product. The *P*_*ENO1*_ overexpression plasmid, pBT1 [80] and the PCR product were then digested using *NotI*, and ligated to create the *P*_*ENO1*_*-FGR41-SAT1* overexpression cassette (pSL003).

The *CWP419* overexpression cassette was created by amplifying a 976bp fragment containing the whole *CWP419* (C1_11990W_A) ORF and 477bp of the 3’UTR immediately following the stop codon with the primer set AKO21 and AKO22, that introduced flanking *NotI* and *SacI* cut sites, respectively. The *P*_*ENO1*_ overexpression plasmid, pBT1 [80], and the PCR product were then digested using *NotI* and *SacI*, and ligated to create the *P*_*ENO1*_*-CWP419-SAT1* overexpression cassette (pAEK001).

The *PGA13* overexpression cassette was created by amplifying a 1,663bp PCR fragment containing the *PGA13* ORF and 291bp of the 3’UTR immediately following the stop codon with the primer set AWO336 and AWO337, that introduced flanking *NotI* and *SacI* cut sites, respectively. The *P*_*ENO1*_ overexpression plasmid, pBT1 [80], and the PCR product were then digested using *NotI* and *SacI*, and ligated to create the *P*_*ENO1*_*-PGA13-SAT1* overexpression cassette (pMM001).

### Strain construction

All *C*. *albicans* deletion strains and missense mutants (S3 Table) were created with the use of CRISPR-Cas9 as previously described [79]. *FGR41* overexpression mutants were generated by digesting the *P*_*ENO1*_*-FGR41-SAT1* plasmid (pSL003) with *MscI* (which cuts a single time within the *ENO1* promoter). The linear plasmid was then purified and transformed into the SC5314 wild-type or *STE11/P*_*tet-off*_*-STE11*^*ΔN467*^ strains via electroporation. Successful transformants were selected for on YPD + 200μg/ml nourseothricin. The same method was then used to create *CWP419* and *PGA13* overexpression mutants in their appropriate strain backgrounds using pAEK001 and pMM001, respectively.

### Immunofluorescent staining, flow cytometry, and microscopy analysis

To stain cell wall components of stationary phase cells, 5ml cultures of *C*. *albicans* strains in YPD were started the morning prior to staining. Cultures were grown shaking at 225 rpm at 30°C for ~8 hours before being back diluted to an OD_600_ of 0.1 in fresh YPD and incubated with shaking at 225rpm at 30°C overnight (~16 hours). To inhibit calcineurin, cells were grown overnight in the presence of either 100μg/ml of cyclosporine A (Torcis) or an appropriate volume of the DMSO solvent control in YPD media. The following morning, cells were diluted to an OD_600_ of 0.5 in PBS and stained to assess ß(1,3)-glucan exposure, total chitin, exposed chitin and mannan as previously described [35,37]. For flow cytometry, a goat-anti-mouse secondary antibody conjugated to R-Phycoerythrin (Jackson Immuno Research) at a 1:300 dilution was used, while a rabbit-anti-mouse IgG secondary antibody conjugated to Alexa Fluor 488 (Jackson Immuno Research) was used at a 1:300 dilution for microscopy.

To assess caspofungin-induced ß(1,3)-glucan exposure, overnight cultures were started as described above and strains were back diluted the following morning to an OD_600_ of 0.1. Cells were then left to grow for 3 hours while shaking at 225 rpm at 30°C. To assess the impact that pharmacological inhibition of calcineurin has on caspofungin-induced unmasking, 100ug/ml of cyclosporine A or an appropriate volume of the DMSO solvent control was added to the media prior to back dilution in the morning. In either control or cyclosporine-treated samples, after 3 hours of incubation, 46.9ng/ml of caspofungin, or an appropriate volume of an ethanol solvent control, was added to each tube and strains were left to incubate while shaking at 225 rpm at 30°C for an additional 30 minutes. Following incubation, 1ml of each culture was removed to assess ß(1,3)-glucan exposure as previously described [35,37].

For all yeast cell staining, ≥3 biological replicates consisting of 100,000 recorded events each, were used to assess all strains analyzed by flow cytometry. Visualization and data analyses were performed in FlowJo (Becton, Dickinson and Company). Statistical significance was determined with the use of a student’s t-test or a one-way ANOVA with Tukey’s post hoc analysis (GraphPad Prism, v7.0c software).

To perform immunofluorescent staining of leukocytes, mouse peripheral blood was collected *via* cardiac puncture into lithium heparin treated collection tubes. 250μL of whole blood were added to 10mL PBS and cells were pelleted at 400x G for 5 minutes. Pelleted cells were depleted of red blood cells (RBCs) by 2 rounds of ACK-lysis. Remaining leukocytes were stained with an antibody cocktail consisting of antibodies against: CD11b (BV421, clone M1/70, BioLegend), CD317 (BV605, clone 927, BioLegend), CD11c (BV785, clone N418, BioLegend), Ly6G (FITC, clone 1A8, BioLegend), Ly6C (PerCP-Cy5.5, clone HK1.4, BioLegend), CD49b (PE, clone DX5, BioLegend), CD45 (AlexaFluor 532, clone 30-F11, ThermoFisher), CD3 (BV510, clone 17A2, BioLegend), CD45R/B220 (BV570, RA3-6B2, BioLegend). Stained cells were fixed and run on a Cytek Northern Lights flow cytometer (Cytek Biosciences) equipped with 405nm and 488nm lasers (Blue, Violet). Visualization and analysis were performed in FlowJo (Becton, Dickinson and Company).

### Comparative analysis of public datasets

Differentially expressed genes identified by Chen *et al*. [36] (genes differentially regulated during hyperactive *STE11*^*ΔN467*^ expression) and Bruno *et al*. [55] (genes differentially regulated during caspofungin exposure), as well as genes identified in a Cytoscape predicted Crz1 regulon created by Ballou *et al*. [29], were pulled and manually collated into a single excel file to identify conserved genes within these datasets. This file was then subsequently used as input for the gdata and VennDiagram packages in RStudio (R Version 1.1.456) for the identification of genes common to all datasets.

### Ethics statement

All animal work used in this study was done under an approved protocol by the University of Tennessee, Knoxville Institution Animal Care and Use Committee (IACUC), and in accordance with the National Institute of Health’s (NIH) ethical guidelines for animal research.

### Mouse model

Outbred ICR mice (ENVIGO) were used for all experiments in this study. To prepare for infection, 50ml cultures of the desired strains were grown overnight in YPD, one day prior to injection. Cells were then prepared as previously described [37] and diluted to either 1x10^7^ cells/ml (for immunocompetent mice) or 1x10^5^ cells/ml (for immunosuppressed mice) in PBS. Mice were then intravenously inoculated via the lateral tail vein with 0.1ml of the appropriate cell suspension. To ensure accurate cell counts, cell viability for each strain was assessed by plating on YPD and allowing cells to grow at 30°C for 24 hours following injection. To assess peripheral leukocyte counts and serum cytokine levels, mice were anesthetized, and peripheral blood was isolated via cardiac puncture in heparin treated tubes (BD Biosciences) prior to being euthanized. To assess kidney fungal burden, mice were euthanized, and their kidneys were harvested and placed into pre-weighed whirl-pack bags (Thermo Fisher Scientific) containing 1ml of water. Each bag was then weighed once more to determine kidney weight and then the kidneys were homogenized. Serial dilutions of the kidney homogenates (10^−1^, 10^−2^, 10^−3^) were created, and 1ml of each dilution was added to 15ml of YPD + 75μg/ml chloramphenicol (Thermo Fisher Scientific) precooled to 55°C for plating. Plates were then left to grow at 30°C for 2 days to determine viable fungal colony forming units per gram of kidney. Once counts were obtained, outliers were determined via an outlier test and distribution normality was assessed with the use of a D’Agostino-Pearson omnibus normality test (GraphPad Prism, v7.0c software). Statistical significance was then determined with the use of either a Mann-Whitney test, a one-way ANOVA with Tukey’s post hoc analysis or a non-parametric Kruskal-Wallis test for data that did not follow a Gaussian distribution (GraphPad Prism, v7.0c software). The number of mice used for each experiment is listed in the figure legend associated with the data, and samples were plated with technical duplicates.

### Immune cell depletion

Non-specific immunosuppression was achieved via recurring injections with cyclophosphamide (Sigma-Aldrich) as previously described [37]. Starting 4 days prior to infection, all mice were weighed and then injected intraperitoneally (I.P.) with 150mg/kg of cyclophosphamide based on their average weights. Immune cell depletion was then maintained by recurring I.P. injections with 150mg/kg of cyclophosphamide every 3 days until the end of the experiment.

### Serum cytokine quantification

Serum cytokine concentrations were determined as previously described [37] using the manufacturer’s protocol for the LEGENDplex Mouse Anti-Virus Response Panel 740622 (Biolegend).

### Hyphal assays

Wild-type, *fgr41Δ/Δ*, and *fgr41Δ/Δ* P_ENO1_-FGR41 strains were grown overnight in 5 mL of YPD while shaking at 225rpm in a 30°C incubator. For hyphal assays in liquid media, overnight cultures were spun down at 3,500x g for 5 minutes and washed twice with 25mL of 1X PBS. Washed cells were diluted to an OD_600_ of 0.1 in 1mL EBSS + 2% FBS + 1% Pen/Strep and further serially diluted 1:10 twice. 100 microliters of the 1 x 10^−2^ dilution was placed into each of three wells of a flat-bottomed 96-well plate and incubated at 37°C + 5% CO_2_. Replicate plates were incubated for one, two, and three hours and after incubation each plate was analyzed using an inverted microscope. Image analysis was performed using ImageJ software (Version 2.0.0). For germination assays, 3 biological replicates were measured for each strain used. At each time point sampled, 6 separate images were taken per sample and all cells (~25–50) within the field of focus were categorized as undifferentiated or a germinated yeast cell. To determine hyphal length, 3 biological replicates with 3 technical replicates for each strain were used. At all time points, 3 separate images were taken per a sample and 10 hyphal cells per an image were measured via pixel length on ImageJ software (Version 2.0.0). Once data were obtained, distribution normality was assessed with the use of a D’Agostino-Pearson omnibus normality test (GraphPad Prism, v7.0c software). Statistical significance was then determined with the use of either a one-way ANOVA with a Tukey’s multiple comparison post-hoc analysis for data that followed a Gaussian distribution or a Kruskal-Wallis test with Dunn’s multiple comparisons post-hoc analysis for data that did not.

### Spot dilution assays and growth curves

To assess the sensitivity of mutants to various cell wall stressors, strains were grown overnight in 5ml cultures in YPD one night prior to the experiment. The following morning, the cultures were harvested and washed 3 times with 5ml of PBS, diluted to an OD_600_ of 0.1 in PBS, and four subsequent 1:10 dilutions were further performed. To assess cell wall sensitivity to cell envelope stressors, 3μl of each dilution was spotted onto a plate consisting of YPD (control), YPD + 10μg/ml calcofluor white (Fisher Scientific), YPD + 50ng/ml or 125ng/ml caspofungin (Gold Biotechnology) or YPD + 0.05% sodium dodecyl sulfate (SDS) (Fisher Scientific). Plates were then incubated for 24 hours at 30°C, and images were taken the following day.

For growth curves, strains were grown overnight in 5ml cultures in YPD one night prior to infection. The following morning, the cultures were diluted to an OD_600_ of 0.1 in 5mls of fresh YPD, and left to grow shaking at 225rpm at 30°C. The OD_600_ was then taken at 2, 4, 6, 8, 10, 24 and 48 hours and growth kinetics were analyzed using GraphPad Prism (v7.0c software). Three biological replicates were tested for each strain. The generation times for each strain were determined from measurements taken during exponential growth phase at 2 and 8 hours post-inoculation.

## Supporting information

S1 Table. Plasmids used in this study (PDF)

S2 Table Primers used in this study. (PDF)

S3 Table *C*. *albicans* strains used in this study. (PDF)

S4 Table Raw values associated with each graph in this study. (XLSX)

S1 Fig Gating strategy for yeast cell population of *C*. *albicans*. (TIF)

S2 Fig Loss of *FGR41* induces changes in total chitin and mannan levels in *C*. *albicans*. (A-C) Overnight cultures of cells were stained with calcofluor white (CFW), fluorescein conjugated wheat germ agglutinin (WGA) and concanavalin A (ConA) to measure total chitin, exposed chitin and mannan levels in the cell wall, respectively. 4 biological replicates were stained for each sample. (A) CFW staining, (B) WGA staining and (C) ConA staining. (****p<0.0001, by one-way ANOVA).
(TIF)

S3 Fig Overexpression of calcineurin effector genes does not restore calcium-induced masking in a *crz1Δ/Δ* mutant. Cells were grown overnight in the absence or presence of 50mM CaCl_2_ in YPD broth. Cells were stained with an anti-ß(1,3)-glucan antibody and a phycoerythrin-conjugated secondary antibody followed by flow cytometry to assess the levels of ß(1,3)-glucan exposure. 3 biological replicates with 1 technical replicate for each were assessed for each strain. (***p<0.001, ****p<0.0001, by one-way ANOVA).
(TIF)

S4 Fig Loss of *FGR41* or *CWP419* does not attenuate fungal growth *in vitro*. Overnight cultures of cells were diluted to an OD_600_ of 0.1 into 5ml of fresh YPD media and left to grow with shaking at 225rpm at 30°C for 48 hours. Cell density was measured every 2 hours for the first 10 hours by measuring OD_600_ and at 24 and 48 hours as well. All samples were run using 3 biological replicates for each strain. (A) *fgr41Δ/Δ* mutant growth curve in YPD. (B) *cwp419Δ/Δ* mutant growth curve in YPD. (C) Generations time for each strain during exponential growth phase.
(TIF)

S5 Fig Characterization of serum cytokines during systemic infection with an *fgr41Δ/Δ* mutant. ICR mice were intravenously infected with 1x10^6^ cells of *C*. *albicans* wild-type (SC5314) or the *fgr41Δ/Δ* mutant. Serum was then collected 4 d.p.i. and cytokines were measured via flow cytometry using the LEGENDplex cytokine bead-based array kit to measure circulatory cytokine levels. (n = 5 mice) (ns = not significant, by Mann-Whitney test).
(TIF)

S6 Fig *FGR41* overexpression does not impact fungal burden 4 days post infection. ICR mice were intravenously infected with 1x10^6^ cells of *C*. *albicans* wild-type (SC5314) or the *P*_*ENO1*_*-FGR41* overexpression strain, and kidneys were subsequently harvested 4 days post infection (d.p.i.) to assess fungal burden. (n = 7–8 mice per strain)(ns = not significant, by Mann-Whitney test).
(TIF)

S7 Fig Loss of *FGR41* modestly delays hyphal development *in vitro*. (A & B) To assess germination frequency and hyphal length, overnight cultures were diluted to an OD_600_ of 0.1 in EBSS + 2% fetal bovine serum and allowed to grow for three hours at 37°C at 5% CO_2_. (A) Samples were measured at each hour to assess germination frequency (n = 3 biological replicates for each strain, with all cells (25–50) counted within 6 separate fields of view for each replicate at all time points sampled). (B) Germinated hyphae were then measured to assess hyphal length. (n = 3 biological replicates with 3 technical replicates for each strain. For each strain, three separate images were taken and 10 hyphal cells for each image were measured using Image J, making an n = 90 for each time point measured). (*p<0.05, **p<0.01, ***p<0.0005, ****p<0.0001 and ns = not significant, via either a one-way ANOVA with Tukey’s multiple comparisons post-hoc analysis or a Kruskal-Wallis test with Dunn’s multiple comparisons post-hoc analysis).
(TIF)

S8 Fig Loss of *FGR41* or *CWP419* does not impact susceptibility to cell wall stressors. Sensitivity phenotypes of the wild-type, *fgr41Δ/Δ*, *cwp419Δ/Δ* and their respective reintegrant controls to 10μg/ml calcofluor white, 50ng/ml and 125ng/ml caspofungin and 0.05% SDS were assessed via a spot dilution assay. (A) Representative images of the growth of the *FGR41* mutants. (B) Representative images of the growth of the *CWP419* mutants.
(TIF)

S9 Fig Cyclophosphamide treatment globally diminishes immune cells at early timepoints. (A) Representative gating strategy used to identify immune cell populations. Leukocytes were stained with antibodies against CD45, Ly6G, Ly6C, CD11b, CD3, CD49b, B220/CD45R, CD317/mPDCA-1, and CD11c. (B) Quantification of respective immune cells in peripheral blood. Mice were treated with either PBS (control) or two recurring injections of 150mg/kg of Cyclophosphamide (Cyclo) (at 4 days prior to infection and 1 day prior to infection). Mice were then further separated into infected (intravenously injected with 1x10^4^ of SC5314 *C*. *albicans* cells) or PBS mock infected control groups and serum was collected 5 hours post infection to assess circulatory leukocyte levels. Immune cells were stratified based on marker expression and cells/μL are shown for each type. CD45+ represents total leukocyte levels. Other cell types examined include neutrophils (Ly6G+/Ly6C+), monocytes (Ly6C+/Ly6G-/CD3-/CD11c-), Dendritic cells (Ly6C+/Ly6G-/CD11c+), NK cells (Ly6G-/Ly6C-/CD3-/CD49b+), NK T cells (Ly6c-/Ly6G-/CD3+/CD49b+), T cells (Ly6G-/Ly6C-/CD3+/CD49b-), Ly6C+ T cells (Ly6G-/Ly6C+/CD3+), and B cells (Ly6G-/Ly6C-/CD45R+). (n = 1 mouse per treatment).
(TIF)
