# Supplementary material for: Cek1 regulates ß(1,3)-glucan exposure through calcineurin effectors in Candida albicans
Source: PLoS Genet. 2022 Sep 19;18(9):e1010405. doi: 10.1371/journal.pgen.1010405 (PMC9521907; doi:10.1371/journal.pgen.1010405)
Supplement: S1 Table. Plasmids used in this study — (PDF) [file pgen.1010405.s001.pdf]

Table S1: Plasmids used in this study.

Strain Name	Description	Marker	Parent/Source
pBT1	*CaNAT-P_{ENO1}*	Ampicillin, Nourseothricin	1
pSL003	*CaNAT-P_{ENO}1-FGR41*	Ampicillin, Nourseothricin	pBT1
pAEK001	*CaNAT- P_{ENO}1-CWP419*	Ampicillin, Nourseothricin	pBT1
pMM001	*CaNAT- P_{ENO}1-PGA13*	Ampicillin, Nourseothricin	pBT1

References

1 Tams, R. N. *et al.* Overproduction of Phospholipids by the Kennedy Pathway Leads to Hypervirulence in Candida albicans. *Front Microbiol* **10**, 86, doi:10.3389/fmicb.2019.00086 (2019).
