# Supplementary material for: Cek1 regulates ß(1,3)-glucan exposure through calcineurin effectors in Candida albicans
Source: PLoS Genet. 2022 Sep 19;18(9):e1010405. doi: 10.1371/journal.pgen.1010405 (PMC9521907; doi:10.1371/journal.pgen.1010405)
Supplement: S2 Table — (PDF) [file pgen.1010405.s002.pdf]

S2 Table: Primers used in this study.

Primer Name	Sequence (5' to 3')	Description	Source
AHO1096	GACGGCACGGCCACGCGTTTAAACCGCC	Fragment A forward primer for CRISPR-mediated gene deletion.	1
AHO1097	CCCGCCAGGCGCTGGGGTTTAAACACCG	Fragment B reverse primer for CRISPR-mediated gene deletion.	1
AHO1098	caaattaaaaatagtttacgcaag	Fragment A reverse primer for CRISPR-mediated gene deletion.	1
AHO1237	aggtgatgctgaagctattgaag	Fragment C forward primer for CRISPR-mediated gene deletion.	1
AHO1238	TGTATTTTGTTTTAAAATTTTAGTGACTGTTTC	Fragment C reverse primer for CRISPR-mediated gene deletion.	1
BTO33	GACTAACGATTTCTATAAGG	P_{ENO1} colony PCR forward check primer and primer for sequencing P_{ENO1} overexpression constructs	2
AWO320	CGTAAACTATTTTTAATTTGATGAAGGTGGATGGACTTTCGTTTTAGAGCTAGAAATAGC	*DFI1* gRNA forward primer for fragment B amplification for CRISPR-mediated gene clevage.	This Study
AWO335	GTTGTTGGTGTAGTTGTTGG	DFI1^WWQQ Sequencing Primer Forward (Sits at +810 and is 101bp upstream of the first missense mutation)	This Study
AWO355	CGTAAACTATTTTTAATTTGcaaataagctggaattaagtGTTTTAGAGCTAGAAATAGC	*FGR41* gRNA forward primer for fragment B	This Study

		amplification for CRISPR-mediated gene deletion.	
AWO359	GAGACAAGAAGTATTATTTCATCTC	*FGR41* whole gene deletion check primer forward (Sits -502bp upstream from start codon in 5'UTR)	This Study
AWO360	CAATTGAAAATTGTGCATG	*FGR41* whole gene deletion check primer reverse (Sits +455bp from stop codon in 3'UTR)	This Study
AWO361	aacttgcggccgcATGAAGATTTCCAACTTAATTCC	*FGR41* forward primer with NotI cutsite (For cloning into pBT1) (Amplifies ORF + 3'UTR with AWO362)	This Study
AWO362	aacttgcggccgcCAAGAAATTCCAAAAGTCTCC	*FGR41* reverse primer with NotI cutsite reverse (For cloning into pBT1) (Sits +270 from stop codon)	This Study
AWO363	GAGAGTGACAGTTGTAGTGATTG	*FGR41* Cloning check primer into pBT1 and sequencing reverse (Use with BTO33). Also used to confirm loss of ORF in deletion mutant.	This Study
AWO378	CGTAAACTATTTTTAATTTGaaataatgataataaaacgaGTTTTAGAGCTAGAAATAGC	*CWP419 (C1_11990W_A)* gRNA forward primer for fragment B amplification for CRISPR-mediated gene deletion.	This Study
AWO382	CTTTGAAAACTGAATTATTGTAAAC	*CWP419 (C1_11990W_A)* whole gene deletion	This Study

		check primer forward (sits -439 from start)	
AWO383	GATGGTGCTAACAATAATATTCC	*CWP419 (C1_11990W_A)* whole gene deletion check primer reverse (sits +318 from stop in 3'UTR)	This Study
AKO021	AAAAGCGGCCGCCCACTCCTACATAATGCTTAG	*CWP419 (C1_11990W_A)* forward primer with NotI cutsite (For cloning into pBT1) (Amplifies ORF + 3'UTR with AKO22)	This Study
AKO022	AAAAGAGCTCTGCCACTAATTTCAAGAAAC	*CWP419 (C1_11990W_A)* reverse primer with SacI cutsite (For cloning into pBT1) (Amplifies ORF + 3'UTR with AKO21)	This Study
AKO023	CCTTCTGGAACTTCAACTTT	*CWP419* Cloning check primer into pBT1 and sequencing reverse (Use with BTO33)	This Study
AWO384	GCTGTTATCATCACTACTTGAGG	*CWP419 (C1_11990W_A)* whole gene deletion check primer reverse (sits +288 from start in ORF)	This Study
SLO015	CGTAAACTATTTTTAATTTGatagctgtgacccatcatccGTTTTAGAGCTAGAAATAGC	*CRZ1* gRNA forward primer for fragment B amplification for CRISPR-mediated gene deletion.	This Study
SLO021	TAATCAAGCTTACCAAGCTATTTTTATTG	*CRZ1* whole gene deletion check primer forward (sits -166bp from start codon in 5'UTR)	This Study
SLO022	TGGCTACTTCTTTTTCTGCGTGTGTGT	*CRZ1* whole gene deletion check primer	This Study

		reverse (sits +305bp from stop codon in 3'UTR)	
SLO023	GAGTACTGCAGCTACGAACAATTCT	*CRZ1* whole gene deletion check primer forward (sits +1205bp from start codon in ORF)	This Study
AWO336	aacttgcggccgcATGAGATCTCCATCACTCG	*PGA13* forward primer with NotI cutsite (For cloning into pBT1) (Amplifies ORF + 3'UTR with AWO337)(Also used for sequencing)	This Study
AWO337	TAAAAgagctCCTTTATAGAAACCAGATCCACC	*PGA13* reverse primer with SacI cutsite (For cloning into pBT1) (Amplifies ORF + 3'UTR with AWO336)	This Study
AWO338	CACATTTGGTGATGGTTAATG	*PGA13* ligation/transformation check primer reverse (Use with BTO33) (Sits +371bp from start codon in ORF)	This Study
AWO339	CCAACCACATTAACCATCAC	*PGA13* sequencing primer forward (sits +363bp in ORF from start)	This Study
AWO340	GGTTGTCACTACTGGTGTCAC	*PGA13* sequencing primer forward (sits +746bp in ORF from start)	This Study
AWO341	CAAGCAGCCATACTACTCAAG	*PGA13* sequencing primer forward (sits	This Study

		+1138bp in ORF from start)	

**CRISPR-Cas9 Repair Templates**			
AWO321	TATTTTACTTGAGAAAGAGAAACAACCGTGATTATGAAGGTGGccaGACTTTCcaGAGAAAGAATGAGAAATTGGGAAGTGATGAGTTCTTCAATGGTGA	Repair template (sense oligomer) for making *DFI1^WWQQ* (Dfi1-calmodulin interaction deficient) mutants. (introduces pt mutations to change W305Q and W308Q, these changes also correspond to the PAM sequences as well to delete create a silent mutation within it in the repair template.) (Check for integration by screening for unique MscI cut site in mutant -> silent mutations for all point mutations introduced).	This Study
AWO322	TCACCATTGAAGAACTCATCACTTCCCAATTTCTCATTCTTTCTCtgGAAAGTCtggCCACCTTCATAATCACGGTTGTTTCTCTTTCTCAAGTAAAATA	Repair template (antisense oligomer) for making *DFI1^WWQQ* (Dfi1-calmodulin interaction deficient) mutants. (introduces pt mutations to change W305Q and W308Q, these changes also correspond to the PAM sequences as well to delete create a silent mutation within it in the repair template.) (Check for integration by screening for unique MscI cut site in mutant -> silent mutations for all point mutations introduced).	This Study
AWO357	TCAACTCAAGTCGTTCCTTTAACAATATCAACATGAAGATTTCCAACTTATGATATGGTGAGTAATGATTGAAAAAAAAAACAACACCCCTTTTAGTTTC	*FGR41* repair template for whole gene deletion (sense oligomer)	This Study
AWO358	GAAACTAAAAGGGGTGTTGTTTTTTTTTTCAATCATTACTCACCATATCATAAGTTGGAAATCTTCATGTTGATATTGTTAAAGGAACGACTTGAGTTGA	*FGR41* repair template for whole gene deletion (antisense oligomer)	This Study
AWO380	TCTACAACTACCACTCCTACATAATGCTTAGTTCTAAAATTTATTCCCTCTAAAGCAAAAAAAAAAAGAATACCTAAGGTTTTGAATTAAACATCAATTT	*CWP419 (C1_11990W_A)* repair template for whole gene deletion (sense oligomer)	This Study
AWO381	AAATTGATGTTTAATTCAAAACCTTAGGTATTCTTTTTTTTTTTGCTTTAGAGGGAATAAATTTTAGAACTAAGCATTATGTAGGAGTGGTAGTTGTAGA	*CWP419 (C1_11990W_A)* repair template for whole gene deletion (antisense oligomer)	This Study
SLO019	TTTATATCTAAATTTCATAAATCCCAATCATGTCTAACAATCCTCATCCCTGATTTGTTTTTTATTTTTGACATTTATACTTTTTTTTTTTTCATATCTT	*CRZ1* repair template for whole gene deletion (sense oligomer)	This Study

SLO020	AAGATATGAAAAAAAAAAAAGTATAAATGTCAAAAATAAAAAACAAATC AGGGATGAGGATTGTTAGACATGATTGGGATTTATGAAATTTAGATATAAA	*CRZ1* repair template for whole gene deletion (antisense oligomer)	This Study

References

1) Nguyen N, Quail MMF, Hernday AD. An Efficient, Rapid, and Recyclable System for CRISPR-Mediated Genome Editing in Candida albicans. mSphere. 2017;2(2). Epub 2017/05/13. doi: 10.1128/mSphereDirect.00149-17. PubMed PMID: 28497115; PubMed Central PMCID: PMCPMC5422035.

2) Tams RN, Cassilly CD, Anaokar S, Brewer WT, Dinsmore JT, Chen YL, et al. Overproduction of Phospholipids by the Kennedy Pathway Leads to Hypervirulence in Candida albicans. Front Microbiol. 2019;10:86. Epub 2019/02/23. doi: 10.3389/fmicb.2019.00086. PubMed PMID: 30792701; PubMed Central PMCID: PMCPMC6374345.
