# Supplementary material for: Cek1 regulates ß(1,3)-glucan exposure through calcineurin effectors in Candida albicans
Source: PLoS Genet. 2022 Sep 19;18(9):e1010405. doi: 10.1371/journal.pgen.1010405 (PMC9521907; doi:10.1371/journal.pgen.1010405)
Supplement: S3 Table — (PDF) [file pgen.1010405.s003.pdf]

Table S3: Strains used in this study.

Strain	Genotype	Parent	Source or Reference
AWY006	*LEU2/leu2Δ* (Wild Type)	SC5314	1
AWY080	*STE11/*$P_{tet\text{-}off}$*-STE11*$^{\Delta N467}$ *LEU2/leu2Δ*	AWY006	1
AWY187	*STE11/*$P_{tet\text{-}off}$*-STE11*$^{\Delta N467}$ *dfi1Δ/Δ LEU2/leu2Δ*	AWY080	1
AWY182	*cph1Δ/Δ LEU2/leu2Δ*	AWY006	1
AWY254	*DFI1*^{W305Q,W308Q}/ *DFI1*^{W305Q,W308Q}	AWY006	This study
AWY255	*STE11/*$P_{tet\text{-}off}$*-STE11*$^{\Delta N467}$ *Dfi1*^{W305Q,W308Q}/ *Dfi1*^{W305Q,W308Q} *LEU2/leu2Δ*	AWY080	This study
SLY020	*crz1Δ/Δ LEU2/leu2Δ*	AWY006	This study
SLY023	*STE11/*$P_{tet\text{-}off}$*-STE11*$^{\Delta N467}$ crz1*Δ/Δ LEU2/leu2Δ*	AWY080	This study
SLY005	*fgr41Δ/Δ LEU2/leu2Δ*	AWY006	This study
SLY007	*STE11/*$P_{tet\text{-}off}$*-STE11*$^{\Delta N467}$ *fgr41Δ/Δ LEU2/leu2Δ*	AWY080	This study
SLY015	P_{ENO1}*-FGR41 LEU2/leu2Δ*	AWY006	This study
SLY013	*fgr41Δ/Δ* P_{ENO1}*-FGR41 LEU2/leu2Δ*	SLY005	This study
SLY017	*STE11/*$P_{tet\text{-}off}$*-STE11*$^{\Delta N467}$ P_{ENO1}*-FGR41 LEU2/leu2Δ*	AWY080	This study
AWY290	*cwp419Δ/Δ LEU2/leu2Δ*	AWY006	This study
AWY292	*STE11/*$P_{tet\text{-}off}$*-STE11*$^{\Delta N467}$ *cwp419Δ/Δ LEU2/leu2Δ*	AWY080	This study
AKY001	P_{ENO1}*-CWP419 LEU2/leu2Δ*	AWY006	This study
AKY004	*STE11/*$P_{tet\text{-}off}$*-STE11*$^{\Delta N467}$ P_{ENO1}*-CWP419 LEU2/leu2Δ*	AWY080	This study
AWY295	*cwp419Δ/Δ* P_{ENO1}*-CWP419 LEU2/leu2Δ*	AWY290	This study
MMY002	P_{ENO1}*-PGA13 LEU2/leu2Δ*	AWY006	This study
MMY003	*STE11/*$P_{tet\text{-}off}$*-STE11*$^{\Delta N467}$ P_{ENO1}*-PGA13 LEU2/leu2Δ*	AWY080	This study
AWY298	*crz1Δ/Δ* P_{ENO1}*-CWP419 LEU2/leu2Δ*	SLY020	This study
AWY302	*crz1Δ/Δ* P_{ENO1}*-FGR41 LEU2/leu2Δ*	SLY020	This study

References:

1 Wagner, A. S. *et al.* Activation of Cph1 causes ss(1,3)-glucan unmasking in Candida albicans and attenuates virulence in mice in a neutrophil-dependent manner. *PLoS Pathog* **17**, e1009839, doi:10.1371/journal.ppat.1009839 (2021).
