# Supplementary figures and images for: Cek1 regulates ß(1,3)-glucan exposure through calcineurin effectors in Candida albicans
Source: PLoS Genet. 2022 Sep 19;18(9):e1010405. doi: 10.1371/journal.pgen.1010405 (PMC9521907; doi:10.1371/journal.pgen.1010405)

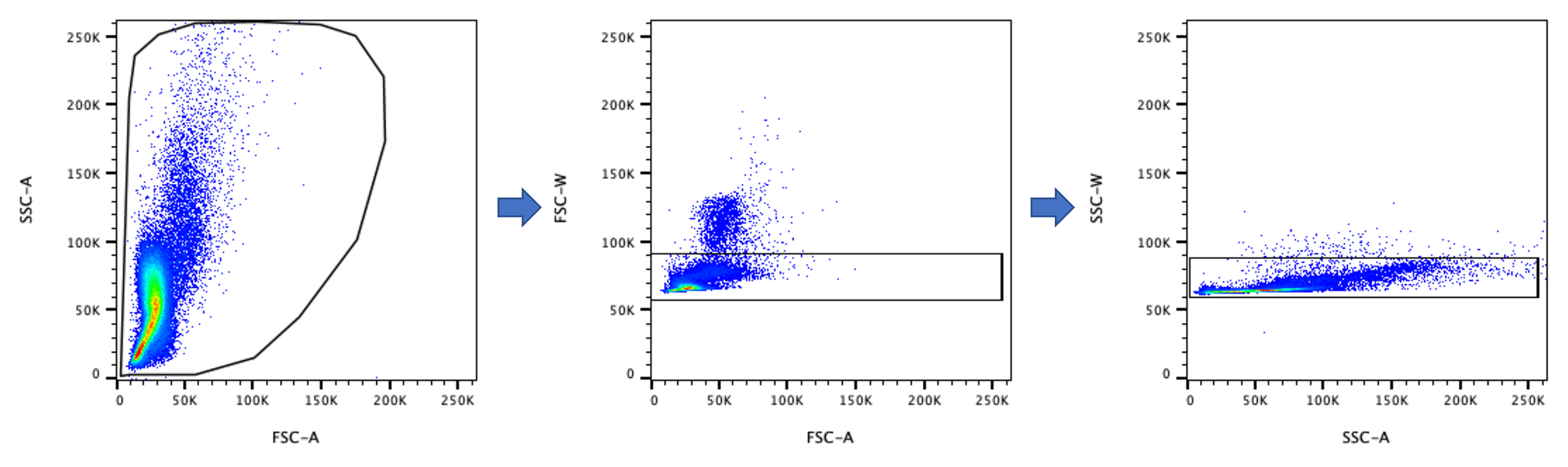

Supplement: S1 Fig — (TIF) [file pgen.1010405.s005.tif]

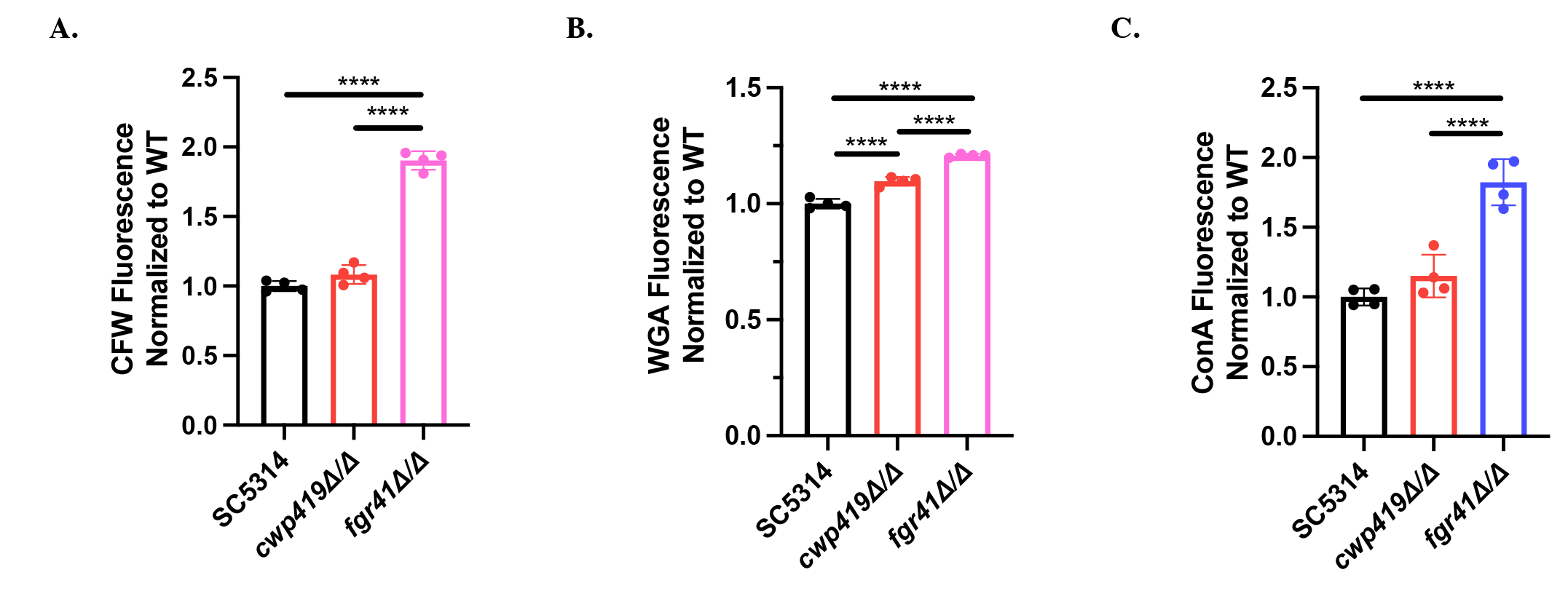

Supplement: S2 Fig — (A-C) Overnight cultures of cells were stained with calcofluor white (CFW), fluorescein conjugated wheat germ agglutinin (WGA) and concanavalin A (ConA) to measure total chitin, exposed chitin and mannan levels in the cell wall, respectively. 4 biological replicates were stained for each sample. (A) CFW staining, (B) WGA staining and (C) ConA staining. (****p<0.0001, by one-way ANOVA). (TIF) [file pgen.1010405.s006.tif]

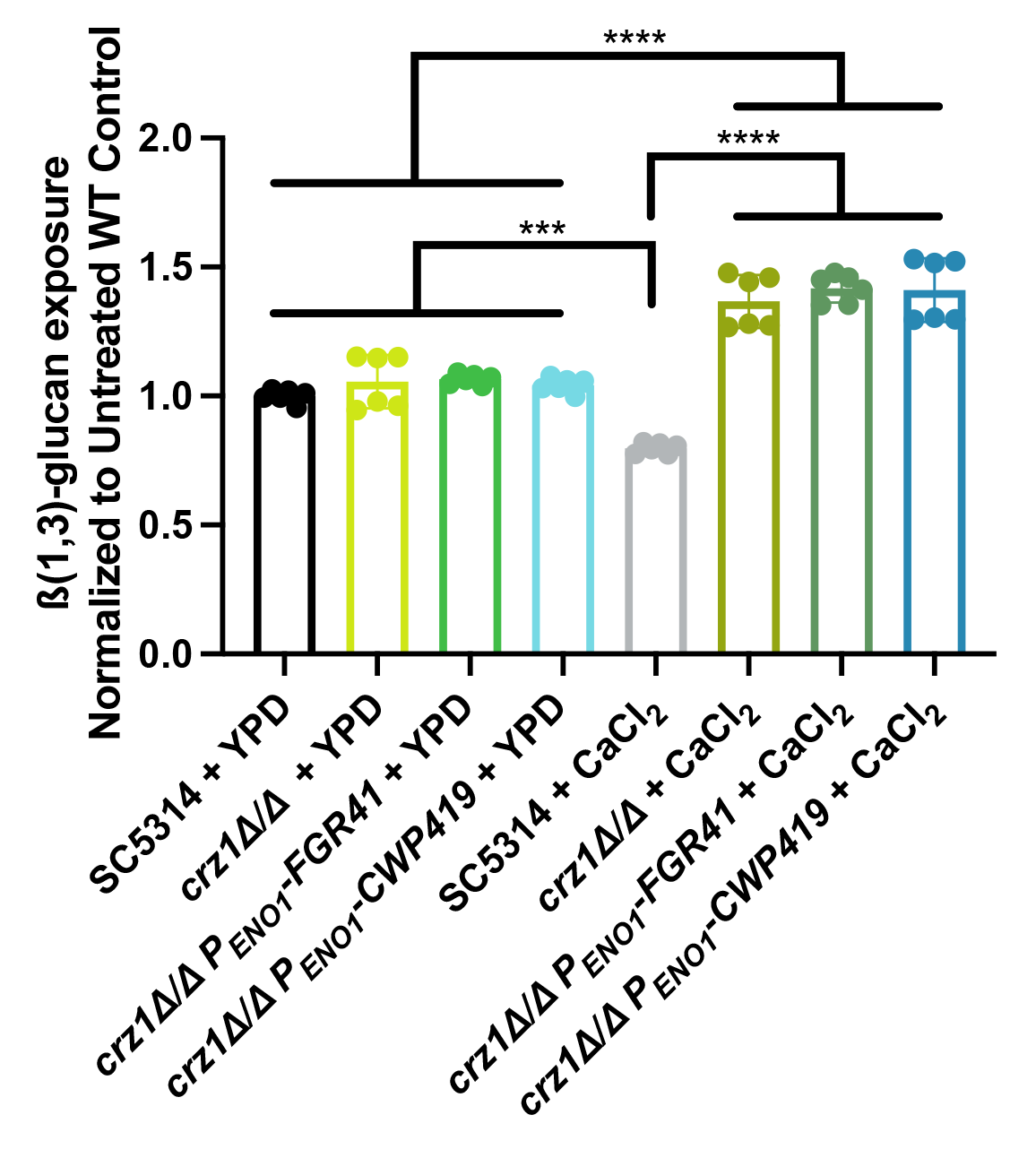

Supplement: S3 Fig — Cells were grown overnight in the absence or presence of 50mM CaCl2 in YPD broth. Cells were stained with an anti-ß(1,3)-glucan antibody and a phycoerythrin-conjugated secondary antibody followed by flow cytometry to assess the levels of ß(1,3)-glucan exposure. 3 biological replicates with 1 technical replicate for each were assessed for each strain. (***p<0.001, ****p<0.0001, by one-way ANOVA). (TIF) [file pgen.1010405.s007.tif]

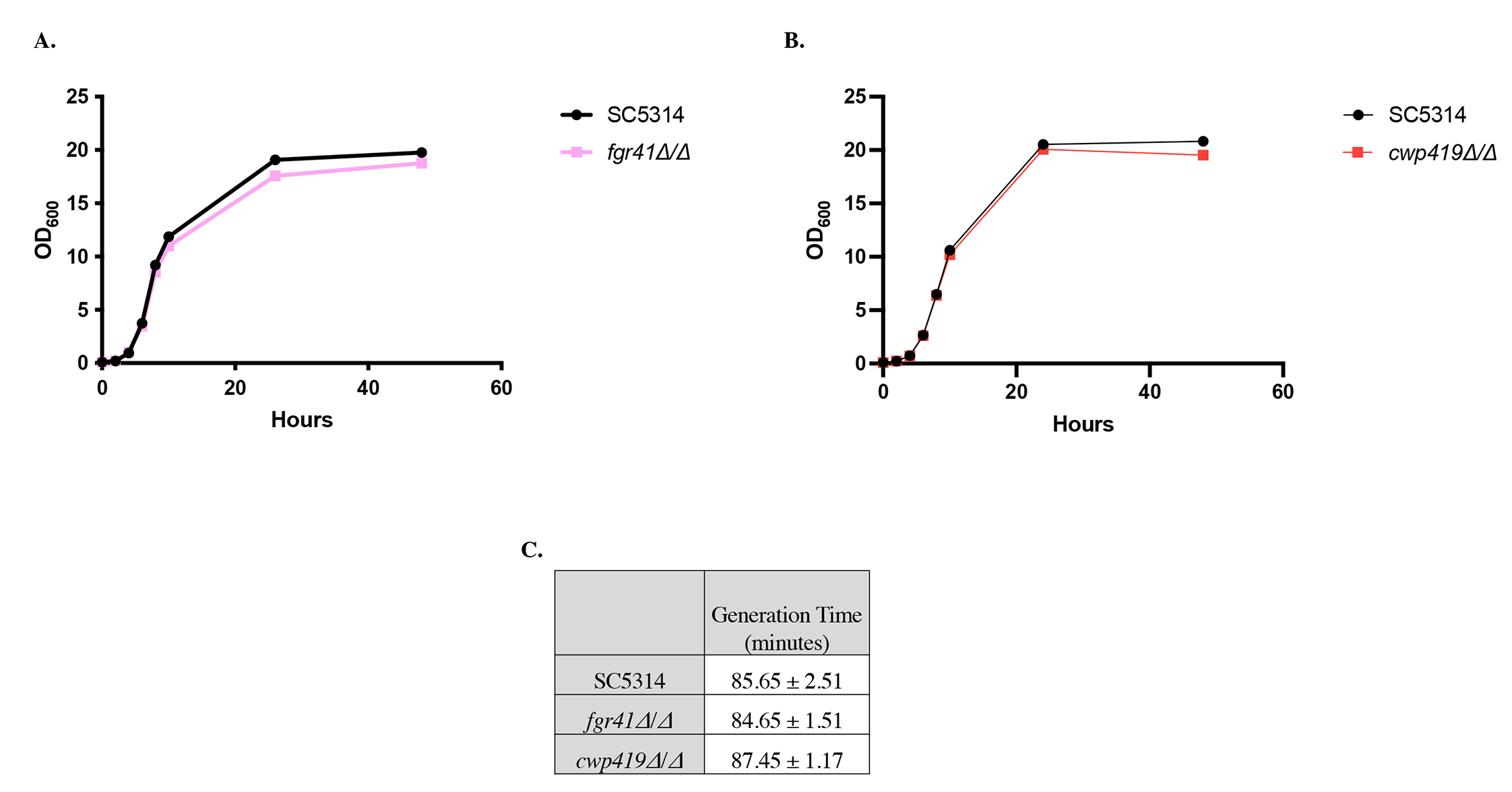

Supplement: S4 Fig — Overnight cultures of cells were diluted to an OD600 of 0.1 into 5ml of fresh YPD media and left to grow with shaking at 225rpm at 30°C for 48 hours. Cell density was measured every 2 hours for the first 10 hours by measuring OD600 and at 24 and 48 hours as well. All samples were run using 3 biological replicates for each strain. (A) fgr41Δ/Δ mutant growth curve in YPD. (B) cwp419Δ/Δ mutant growth curve in YPD. (C) Generations time for each strain during exponential growth phase. (TIF) [file pgen.1010405.s008.tif]

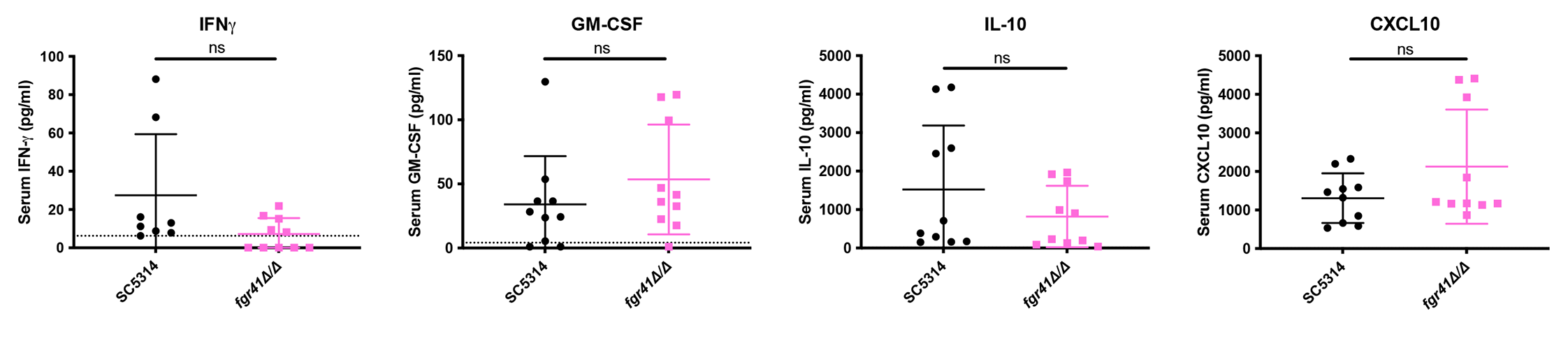

Supplement: S5 Fig — ICR mice were intravenously infected with 1x10^6^ cells of C. albicans wild-type (SC5314) or the fgr41Δ/Δ mutant. Serum was then collected 4 d.p.i. and cytokines were measured via flow cytometry using the LEGENDplex cytokine bead-based array kit to measure circulatory cytokine levels. (n = 5 mice) (ns = not significant, by Mann-Whitney test). (TIF) [file pgen.1010405.s009.tif]

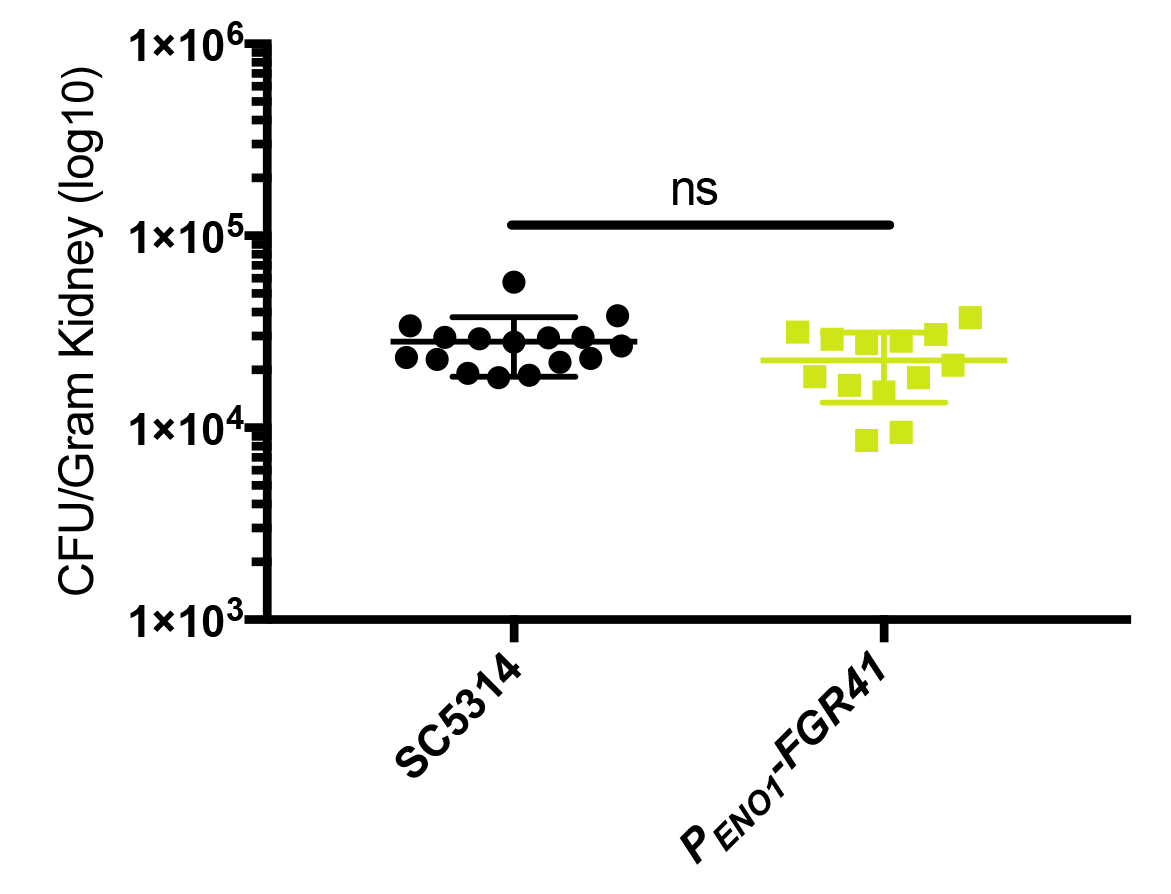

Supplement: S6 Fig — ICR mice were intravenously infected with 1x10^6^ cells of C. albicans wild-type (SC5314) or the PENO1-FGR41 overexpression strain, and kidneys were subsequently harvested 4 days post infection (d.p.i.) to assess fungal burden. (n = 7–8 mice per strain)(ns = not significant, by Mann-Whitney test). (TIF) [file pgen.1010405.s010.tif]

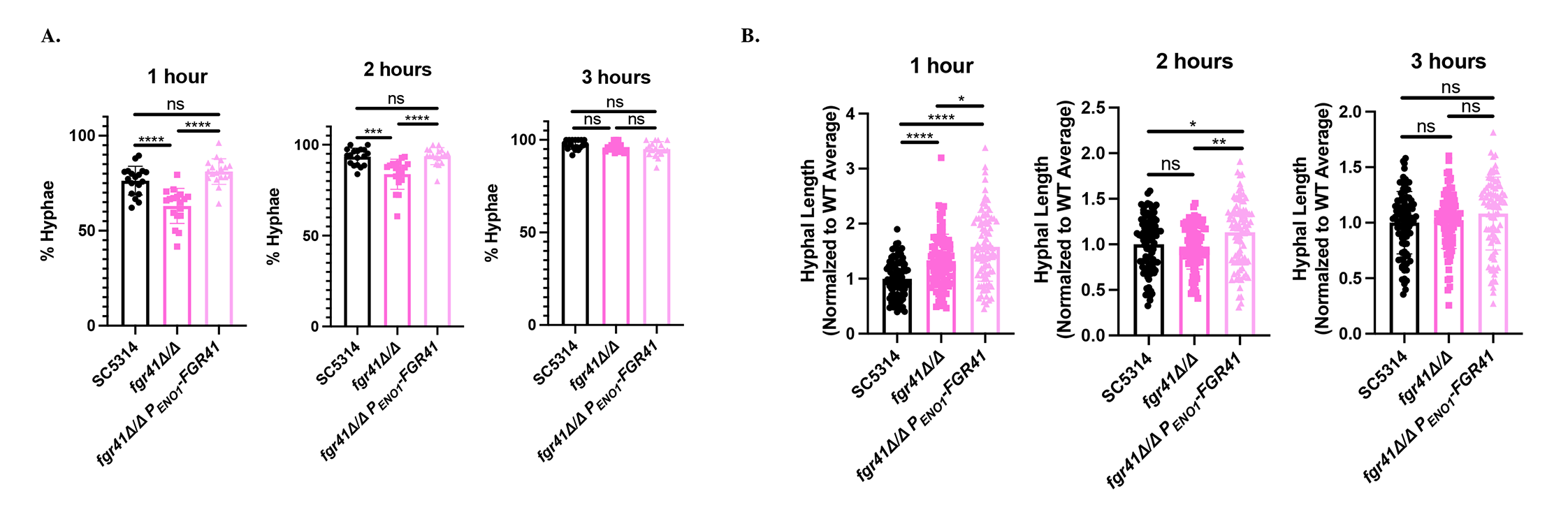

Supplement: S7 Fig — (A & B) To assess germination frequency and hyphal length, overnight cultures were diluted to an OD600 of 0.1 in EBSS + 2% fetal bovine serum and allowed to grow for three hours at 37°C at 5% CO2. (A) Samples were measured at each hour to assess germination frequency (n = 3 biological replicates for each strain, with all cells (25–50) counted within 6 separate fields of view for each replicate at all time points sampled). (B) Germinated hyphae were then measured to assess hyphal length. (n = 3 biological replicates with 3 technical replicates for each strain. For each strain, three separate images were taken and 10 hyphal cells for each image were measured using Image J, making an n = 90 for each time point measured). (*p<0.05, **p<0.01, ***p<0.0005, ****p<0.0001 and ns = not significant, via either a one-way ANOVA with Tukey’s multiple comparisons post-hoc analysis or a Kruskal-Wallis test with Dunn’s multiple comparisons post-hoc analysis). (TIF) [file pgen.1010405.s011.tif]

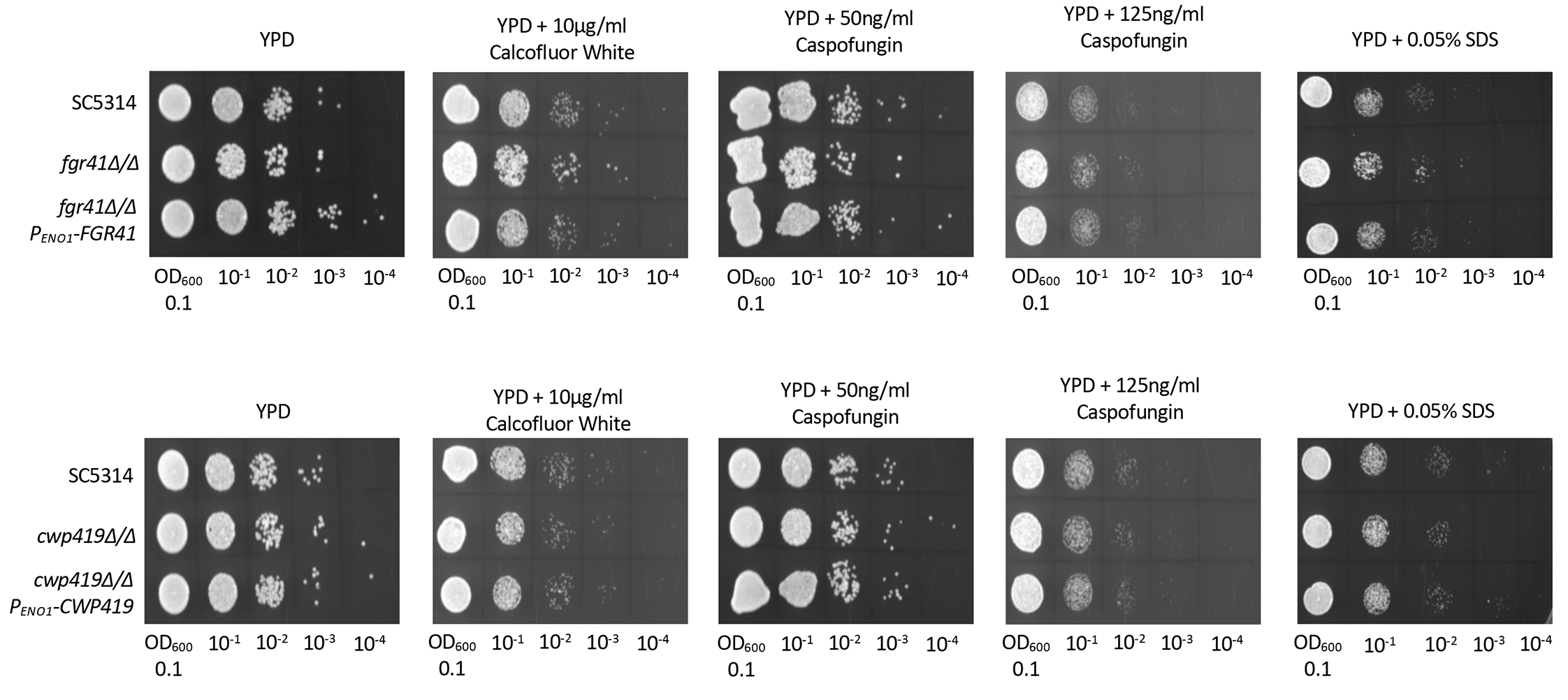

Supplement: S8 Fig — Sensitivity phenotypes of the wild-type, fgr41Δ/Δ, cwp419Δ/Δ and their respective reintegrant controls to 10μg/ml calcofluor white, 50ng/ml and 125ng/ml caspofungin and 0.05% SDS were assessed via a spot dilution assay. (A) Representative images of the growth of the FGR41 mutants. (B) Representative images of the growth of the CWP419 mutants. (TIF) [file pgen.1010405.s012.tif]

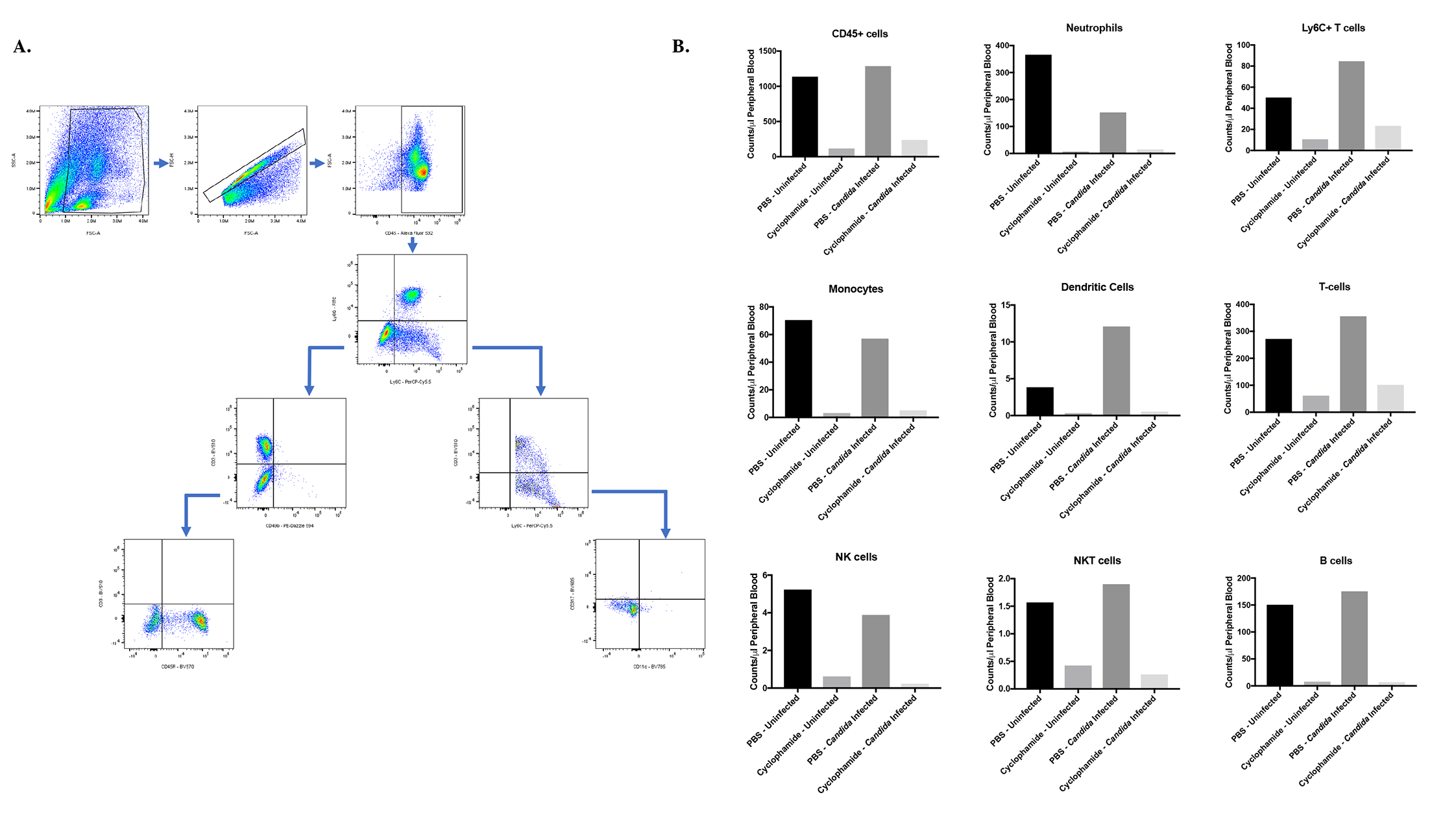

Supplement: S9 Fig — (A) Representative gating strategy used to identify immune cell populations. Leukocytes were stained with antibodies against CD45, Ly6G, Ly6C, CD11b, CD3, CD49b, B220/CD45R, CD317/mPDCA-1, and CD11c. (B) Quantification of respective immune cells in peripheral blood. Mice were treated with either PBS (control) or two recurring injections of 150mg/kg of Cyclophosphamide (Cyclo) (at 4 days prior to infection and 1 day prior to infection). Mice were then further separated into infected (intravenously injected with 1x10^4^ of SC5314 C. albicans cells) or PBS mock infected control groups and serum was collected 5 hours post infection to assess circulatory leukocyte levels. Immune cells were stratified based on marker expression and cells/μL are shown for each type. CD45+ represents total leukocyte levels. Other cell types examined include neutrophils (Ly6G+/Ly6C+), monocytes (Ly6C+/Ly6G-/CD3-/CD11c-), Dendritic cells (Ly6C+/Ly6G-/CD11c+), NK cells (Ly6G-/Ly6C-/CD3-/CD49b+), NK T cells (Ly6c-/Ly6G-/CD3+/CD49b+), T cells (Ly6G-/Ly6C-/CD3+/CD49b-), Ly6C+ T cells (Ly6G-/Ly6C+/CD3+), and B cells (Ly6G-/Ly6C-/CD45R+). (n = 1 mouse per treatment). (TIF) [file pgen.1010405.s013.tif]
